# Fine mapping the *CETP* region reveals a common intronic insertion associated to HDL-C

**DOI:** 10.1038/npjamd.2015.11

**Published:** 2015-11-12

**Authors:** Elisabeth M van Leeuwen, Jennifer E Huffman, Joshua C Bis, Aaron Isaacs, Monique Mulder, Aniko Sabo, Albert V Smith, Serkalem Demissie, Ani Manichaikul, Jennifer A Brody, Mary F Feitosa, Qing Duan, Katharina E Schraut, Pau Navarro, Jana V van Vliet-Ostaptchouk, Gu Zhu, Hamdi Mbarek, Stella Trompet, Niek Verweij, Leo-Pekka Lyytikäinen, Joris Deelen, Ilja M Nolte, Sander W van der Laan, Gail Davies, Andrea JM Vermeij-Verdoold, Andy ALJ van Oosterhout, Jeannette M Vergeer-Drop, Dan E Arking, Holly Trochet, Generation Scotland, Carolina Medina-Gomez, Fernando Rivadeneira, Andre G Uitterlinden, Abbas Dehghan, Oscar H Franco, Eric J Sijbrands, Albert Hofman, Charles C White, Josyf C Mychaleckyj, Gina M Peloso, Morris A Swertz, LifeLines Cohort Study, Gonneke Willemsen, Eco J de Geus, Yuri Milaneschi, Brenda WJH Penninx, Ian Ford, Brendan M Buckley, Anton JM de Craen, John M Starr, Ian J Deary, Gerard Pasterkamp, Albertine J Oldehinkel, Harold Snieder, P Eline Slagboom, Kjell Nikus, Mika Kähönen, Terho Lehtimäki, Jorma S Viikari, Olli T Raitakari, Pim van der Harst, J Wouter Jukema, Jouke-Jan Hottenga, Dorret I Boomsma, John B Whitfield, Grant Montgomery, Nicholas G Martin, CHARGE Lipids Working Group, Ozren Polasek, Veronique Vitart, Caroline Hayward, Ivana Kolcic, Alan F Wright, Igor Rudan, Peter K Joshi, James F Wilson, Leslie A Lange, James G Wilson, Vilmundur Gudnason, Tamar B Harris, Alanna C Morrison, Ingrid B Borecki, Stephen S Rich, Sandosh Padmanabhan, Bruce M Psaty, Jerome I Rotter, Blair H Smith, Eric Boerwinkle, L Adrienne Cupples, Cornelia van Duijn

**Affiliations:** 1 Department of Epidemiology, Erasmus Medical Center, Rotterdam, The Netherlands; 2 MRC Human Genetics Unit, MRC IGMM, University of Edinburgh, Edinburgh, UK; 3 National Heart, Lung, and Blood Institute (NHLBI) Cardiovascular Epidemiology and Human Genomics Branch, Framingham Heart Study, Framingham, MA, USA; 4 Department of Medicine, University of Washington, Seattle, WA, USA; 5 Department of Internal Medicine, Erasmus Medical Center, Rotterdam, The Netherlands; 6 Human Genome Sequencing Center, Baylor College of Medicine, Houston, TX, USA; 7 Icelandic Heart Association, Kopavogur, Iceland; 8 Faculty of Medicine, University of Iceland, Reykjavik, Iceland; 9 Department of Biostatistics, Boston University School of Public Health, Boston, MA, USA; 10 Center for Public Health Genomics, Department of Public Health Sciences, University of Virginia, Charlottesville, VA, USA; 11 Department of Genetics, Washington University School of Medicine, St Louis, MO, USA; 12 Department of Genetics, University of North Carolina, Chapel Hill, NC, USA; 13 Centre for Population Health Sciences, University of Edinburgh, Edinburgh, Scotland; 14 Department of Endocrinology, University of Groningen, University Medical Center Groningen, Groningen, The Netherlands; 15 Genetic Epidemiology, QIMR Berghofer Medical Research Institute, Brisbane, QLD, Australia; 16 Department of Biological Psychology, VU University Amsterdam and EMGO Institute for Health and Care Research, Amsterdam, The Netherlands; 17 Department of Cardiology, Leiden University Medical Center, Leiden, The Netherlands; 18 Department of Gerontology and Geriatrics, Leiden University Medical Center, Leiden, The Netherlands; 19 Department of Cardiology, University of Groningen, University Medical Center Groningen, Groningen, The Netherlands; 20 Department of Clinical Chemistry, Fimlab Laboratories and University of Tampere School of Medicine, Tampere, Finland; 21 Department of Molecular Epidemiology, Leiden University Medical Center, Leiden, The Netherlands; 22 Department of Epidemiology, University of Groningen, University Medical Center Groningen, Groningen, The Netherlands; 23 Department of Experimental Cardiology, UMC Utrecht, Utrecht, The Netherlands; 24 Centre for Cognitive Ageing and Cognitive Epidemiology, University of Edinburgh, Edinburgh, UK; 25 Department of Psychology, University of Edinburgh, Edinburgh, UK; 26 McKusick-Nathans Institute of Genetic Medicine, Johns Hopkins University School of Medicine, Baltimore, MD, USA; 27 Program in Translational NeuroPsychiatric Genomics, Institute for the Neurosciences, Departments of Neurology and Psychiatry, Brigham and Women’s Hospital, Boston, MA, USA; 28 Program in Medical and Population Genetics, Broad Institute, Cambridge, MA, USA; 29 Ann Romney Center for Neurologic Diseases, Brigham and Women's Hospital, Boston, MA, USA; 30 Center for Human Genetic Research, Massachusetts General Hospital, Boston, MA, USA; 31 Cardiovascular Research Center, Massachusetts General Hospital, Boston, MA, USA; 32 Harvard Medical School, Boston, MA, USA; 33 Department of Genetics, University of Groningen, University Medical Center Groningen, Groningen, The Netherlands; 34 Department of Psychiatry, VU University Medical Center Amsterdam/GGZinGeest and EMGO Institute for Health and Care Research and Neuroscience Campus Amsterdam, Amsterdam, The Netherlands; 35 Robertson Center for Biostatistics, University of Glasgow, Glasgow, UK; 36 Department of Pharmacology and Therapeutics, University College Cork, Cork, Ireland; 37 Alzheimer Scotland Dementia Research Centre, University of Edinburgh, Edinburgh, UK; 38 Laboratory of Clinical Chemistry and Hematology, Division Laboratories & Pharmacy, UMC Utrecht, Utrecht, the Netherlands; 39 Interdisciplinary Center Psychopathology and Emotion Regulation, University of Groningen, University Medical Center Groningen, Groningen, The Netherlands; 40 Department of Cardiology, Heart Centre, Tampere University Hospital and University of Tampere School of Medicine, Tampere, Finland; 41 Department of Clinical Physiology, Tampere University Hospital and University of Tampere School of Medicine, Tampere, Finland; 42 Division of Medicine, Turku University Hospital, and Department of Medicine, University of Turku, Turku, Finland; 43 Department of Clinical Physiology and Nuclear Medicine, Turku University Hospital, and Research Centre of Applied and Preventive Cardiovascular Medicine, University of Turku, Turku, Finland; 44 Molecular Epidemiology, QIMR Berghofer Medical Research Institute, Brisbane, QLD, Australia; 45 Department of Public Health, Faculty of Medicine, University of Split, Split, Croatia; 46 Centre for Population Health Sciences, Medical School, University of Edinburgh, Edinburgh, UK; 47 Department of Physiology and Biophysics, University of Mississippi Medical Center, Jackson, MS, USA; 48 National Institute on Aging, National Institute of Health, Bethesda, MD, USA; 49 Human Genetics Center, The University of Texas School of Public Health, Houston, TX, USA; 50 Division of Cardiovascular and Medical Sciences, University of Glasgow, Glasgow, UK; 51 Department of Medicine, Epidemiology & Health Services, University of Washington, Seattle, WA, USA; 52 Group Health Research Institute, Group Health cooperative, Seattle, WA, USA; 53 Institute for Translational Genomics and Population Sciences, Los Angeles BioMedical Research Institute at Harbor-UCLA Medical Center, Torrance, CA, USA; 54 Division of Genomic Outcomes, Departments of Pediatrics and Medicine, Harbor-UCLA Medical Center, Torrance, CA, USA; 55 Departments of Pediatrics, Medicine, and Human Genetics, UCLA, Los Angeles, CA, USA; 56 Medical Research Institute, University of Dundee, Dundee, UK; 57 Framingham Heart Study, Framingham, MA, USA

## Abstract

**Background:**

Individuals with exceptional longevity and their offspring have significantly larger high-density lipoprotein concentrations (HDL-C) particle sizes due to the increased homozygosity for the I405V variant in the cholesteryl ester transfer protein (*CETP)* gene. In this study, we investigate the association of *CETP* and HDL-C further to identify novel, independent *CETP* variants associated with HDL-C in humans.

**Methods:**

We performed a meta-analysis of HDL-C within the *CETP* region using 59,432 individuals imputed with 1000 Genomes data. We performed replication in an independent sample of 47,866 individuals and validation was done by Sanger sequencing.

**Results:**

The meta-analysis of HDL-C within the *CETP* region identified five independent variants, including an exonic variant and a common intronic insertion. We replicated these 5 variants significantly in an independent sample of 47,866 individuals. Sanger sequencing of the insertion within a single family confirmed segregation of this variant. The strongest reported association between HDL-C and *CETP* variants, was rs3764261; however, after conditioning on the five novel variants we identified the support for rs3764261 was highly reduced (*β*_unadjusted_=3.179 mg/dl (*P* value=5.25×10^−509^), *β*_adjusted_=0.859 mg/dl (*P* value=9.51×10^−25^)), and this finding suggests that these five novel variants may partly explain the association of *CETP* with HDL-C. Indeed, three of the five novel variants (rs34065661, rs5817082, rs7499892) are independent of rs3764261.

**Conclusions:**

The causal variants in *CETP* that account for the association with HDL-C remain unknown. We used studies imputed to the 1000 Genomes reference panel for fine mapping of the *CETP* region. We identified and validated five variants within this region that may partly account for the association of the known variant (rs3764261), as well as other sources of genetic contribution to HDL-C.

## Introduction

Aging is characterized by a deterioration in the maintenance of homeostatic processes over time, leading to functional decline and increased risk for disease and death.^1^ One of the genes linked to healthy aging and longevity is the cholesteryl ester transfer protein (*CETP*) gene.^1,2^ Homozygosity in the 405VV variants of *CETP* is associated with lower concentrations of *CETP*, higher concentrations of high-density lipoprotein concentrations (HDL-C), and greater HDL-C particle size, all associated with both protection against cardiovascular disease^3^ and exceptional longevity.^4^

Functional analyses in mice,^5^ hamsters,^6^ and rabbits^7^ have revealed that the protein encoded by the *CETP* gene mediates the transfer of cholesteryl esters from HDL-C to other lipoproteins such as atherogenic (V)LDL particle and is a key participant in the reverse transport of cholesterol from the periphery to the liver.^8^ Due to the function of *CETP* and the association of the gene with HDL-C in humans,^9,10^ the *CETP* gene is one of the targets for drug development for dyslipidemia.^6,11,12^ *CETP*-inhibition leads to an increase of HDL-C from 30 up to 140% depending on the compound used. The first drug of its class, Torcetrapib was unfortunately associated with an increased mortality and morbidity in patients receiving the *CETP* inhibitor in addition to atorvastatin.^13,14^

The estimated heritability of HDL-C levels is high in humans: 47–76%.^15–23^ Previously published whole-genome sequence data^23^ reported that common variants (minor allele frequency (MAF)>1%) explain up to 61.8% of the variance in HDL-C levels and that rare variants (MAF<1%) explain an additional 7.8% of the variance. Genome-wide association studies revealed that numerous variants are associated with HDL-C, among which are various common^9,10^ and rare^24,25^ variants within the *CETP* gene in multiple ancestries.^4,8,26–28^ In this paper, we investigate the association between *CETP* and HDL-C in humans in further detail to identify variants that are likely to be causal.

To this end, we used a meta-analysis of association studies with imputed genotypes within the *CETP* region. Our study consisted of data from 59,432 samples, of which the genotypes were imputed to the 1000 Genomes project reference panel (version Phase 1 integrated release v3, April 2012, all populations). By using 1000 Genomes imputed data, we expected to find more rare or low-frequent variants, as well as novel insertions and deletions.

## Materials and Methods

### Study descriptions

The descriptions of the participating cohorts can be found in the Supplementary Material. All studies were performed with the approval of the local medical ethics committees, and written informed consent was obtained from all participants.

### Study samples and phenotypes

The total number of individuals in the discovery phase was 59,432 and in the replication phase 47,866. Of the discovery samples, 44,108 individuals (74.21%) were of European ancestry. Of the replication samples, 47,081 individuals (98.36%) were of European ancestry. A summary of the details of both the discovery and replication cohorts participating in this study can be found in Supplementary Table 1.

### Genotyping and imputations

All cohorts were genotyped using commercially available Affymetrix or Illumina genotyping arrays, or custom Perlegen arrays. Quality control was performed independently for each study. To facilitate meta-analysis and replication, each discovery and replication cohort performed genotype imputation using IMPUTE2^29^ or Minimac^30^ with reference to the 1000 Genomes project reference panel. The details per cohort can be found in Supplementary Table 2.

### Association analysis in discovery cohorts

The lipid measurements were adjusted for sex, age, and age^2^ in all cohorts, and if necessary also for cohort-specific covariates (Supplementary Table 1). Some cohorts included samples using lipid-lowering medication; we did not adjust for lipid-lowering medication in our analysis because HDL-C levels are only minimally influenced by lipid-lowering medication. Each discovery cohort ran association analysis for all variants within the *CETP* region (chromosome 16, 56.99–57.02 Mbp) with HDL-C.

### Meta-analysis of discovery cohorts

The association results of all discovery cohorts for all variants within the *CETP* region (chromosome 16, 56.99–57.02 Mbp) were combined using inverse-variance weighting as applied by METAL.^31^ This tool also applies genomic control by automatically correcting the test statistics to account for small amounts of population stratification or unaccounted relatedness and the tool also allows for heterogeneity. We used the following filters for the variants: 0.3<*R*^2^ (measurement for the imputation quality)<1.0 and expected minor allele count (expMAC=2×MAF×*R*^2^×sample size)>10 prior to meta-analysis. After meta-analysis of all available variants, we excluded the variants that were not present in at least three cohorts, to prevent false positive findings.

### Selection of independent variants

To select only variants that were independently associated with HDL-C, we used the Genome-wide Complex Trait Analysis (GCTA) tool, version 1.13.^32^ Although this tool currently supports multiple functionalities, we only used the functions for conditional and joint genome-wide association analysis. This function performs a stepwise selection procedure to select independent single nucleotide polymorphisms (SNP) associations by a conditional and joint analysis approach. It utilizes summary-level statistics from the meta-analysis and linkage disequilibrium (LD) corrections between SNPs are estimated from the 1000 Genomes (1000G Phase I Integrated Release Version 22 Haplotypes (2010–11 data freeze, 14 February 2012 haplotypes)). GCTA estimates the effective sample size and determines the effect size, the s.e., and the *P* value from a joint analysis of all the selected SNPs. In this way, we select the best associated variants in *CETP*. We subsequently checked whether these variants were in LD within the 1000 Genomes reference panel using PLINK^33^ software (Supplementary Table 3).

### Replication of independent CETP variants

Five variants were selected for replication in a sample of 12 independent cohorts: Athero-Express, CHS, FINCAVAS, LBC1936, Lifelines, LLS, NTR-NESDA, PREVEND, PROSPER, QIMR, TRAILS, and YFS. The lipid measurements were adjusted for sex, age, and age^2^ in all cohorts, and if necessary also for cohort-specific covariates (Supplementary Table 1b). The details per cohort regarding variant genotyping and imputations can be found in Supplementary Table 2. The association results of all replication cohorts were combined and the s.e.-based weights were calculated by METAL.^31^ Since none of the five variants are in LD (Supplementary Table 3), the Bonferroni-corrected *P* value for multiple testing was 0.01.

### Test previous published results

The meta-analysis of HDL-C as published by Teslovich *et al.*^9^ identified 38 genome-wide significant (*P* value<5×10^−8^) variants within the *CETP* region (chromosome 16, 56.99–57.02 Mbp). Within all discovery and replication cohorts, we tested these 38 variants, adjusting for the 5 newly identified independent variants to explore whether the new variants explain previously published results. The association results of all cohorts were combined and the s.e.-based weights were calculated by METAL.^31^

We used the genotypes of all 1,092 individuals of the 1000 Genomes project to calculate the correlation between the 38 variants. This correlation matrix was used by matSpDlite^34^ which examines the ratio of observed eigenvalue variance to its theoretical maximum to determine the number of independent variables. For these 38 genome-wide significant variants within the *CETP* region, the effective number of independent variables is 18 and therefore the experiment-wide significance threshold required to keep type I error rate at 5% is 2.85×10^−3^.

### Conditional analysis of independent CETP variants

The replicated independent variants were selected for conditional analysis in both the discovery and the replication cohorts. In this analysis we adjusted for the lead SNP for this region as reported by Teslovich *et al.*^9^ (rs3764261, chromosome 16, position 56,993,324 bp). The association results of all discovery and replication cohorts were combined and the s.e. based weights were calculated by METAL.^31^ The Bonferroni-corrected *P* value for multiple testing was 0.01, since none of the five variants is in LD (Supplementary Table 3).

### Validation of the new CETP insertion within a family

Within the ERF study, 3,658 individuals have been genotyped on various Illumina (Illumina, San Diego, CA, USA) and Affymetrix chips (Affymetrix, Santa Clara, CA, USA), followed by imputations with MaCH (1.0.18c) and Minimac (minimac-β-14 March 2012) to the 1000 Genomes reference panel. Based on the best guess imputed genotypes, we selected one family in which we expected the insertion to segregate.

Validation of the insertion was performed by Sanger sequencing. Genomic DNA was isolated from peripheral blood using standard protocols (salting-out). The intron 2–3 of the *CETP* gene (Supplementary Table 4) was amplified using PCR and the following primer sequences were used to amplify: forward; 5ʹ-tgggggactcaggtctctcc-3ʹ; reverse; 5ʹ-aaagcacctggcccacaacc-3ʹ; size 409 bp.

PCR reactions was performed in 17.5 μl containing 37.5 ng DNA, 10 pmol/μl of each primer, 2.5 mM dNTPs, 10x PCR buffer with Mg^+^ (Roche) and 5 U/μl FastStart Taq (Roche Nederland B.V., Woerden, the Netherlands). Cycle conditions: 7 min at 94 °C; 10 cycles of 30-s denaturation at 94 °C, 30 s annealing at 70 –1 °C per cycle and 90-s extension at 72 °C; followed by 20 cycles of 30-s denaturation at 94 °C, 30 s at 60 °C, and 90 s at 72 °C; final extension 10 min at 72 °C. Sephadex G50 (Amersham Biosciences) was used to purify the sequenced PCR products. Direct sequencing of both strands was performed using Big Dye Terminator chemistry version 4 (Applied Biosystems, Bleiswijk, the Netherlands). Fragments were loaded on an ABI3100 automated sequencer and analyzed with DNA Sequencing Analysis (version 5.3) and SeqScape (version 2.6) software (Applied Biosystems). All sequence variants are numbered at the nucleotide levels according to the following references: NC_000016.10:g.56963437_56963438insA (NCBI), NM_000078.2:c.233+313_233+314insA, Human Feb. 2009 (GRCh37/hg19) Assembly.

## Results

### Meta-analysis in all discovery cohorts to select independent variants

**Figure 1:**
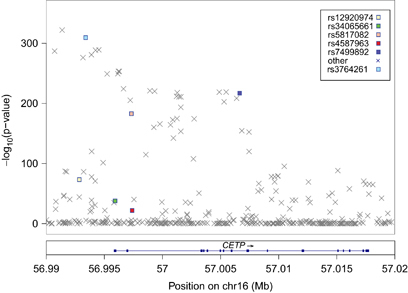
Results of the meta-analysis of all discovery cohorts within the *CETP* region. *CETP*, cholesteryl ester transfer protein.

**Figure 2:**
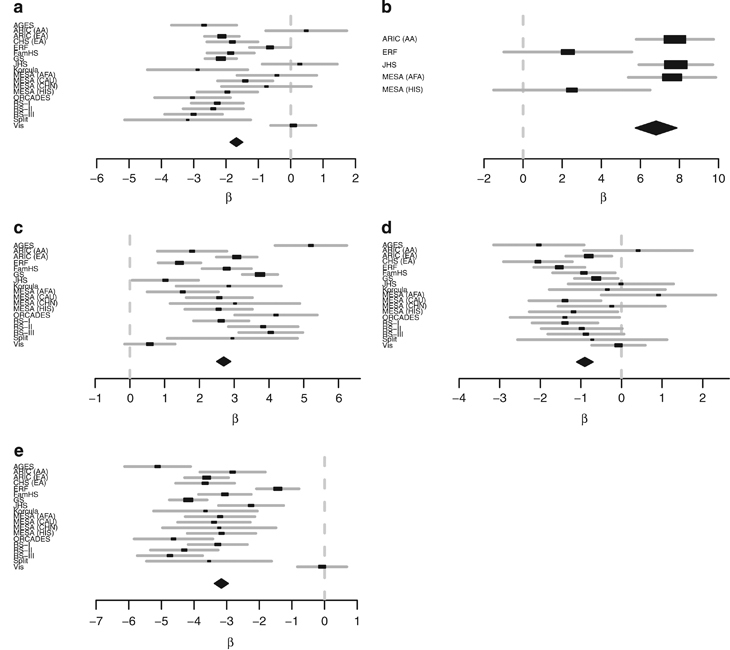
Forest plots from the discovery meta-analysis results for the five independent variants identified within the *CETP* region. Only cohorts in which the variants passed QC are included in the forest plot. (**a**) rs12920974 (chromosome 16, position 56,993,025), (**b**) rs34065661 (chromosome 16, position 56,995,935), (**c**) rs5817082 (chromosome 16, position 56,997,349), (**d**) rs4587963 (chromosome 16, position 56,997,369), and (**e**) rs7499892 (chromosome 16, position 57,006,590). *CETP*, cholesteryl ester transfer protein.

The association of all variants within the *CETP* region (chromosome 16, 56.99–57.02 Mbp) to HDL-C was tested in all discovery cohorts. These results were combined using the inverse-variance weights as applied by METAL.^31^ After exclusion of the variants that were not present in at least 3 cohorts, 254 variants remained (Figure 1). A conditional and joint analysis of the 254 variants using GCTA identified 5 independent variants (Figure 2). Three variants were intronic (rs5817082, rs4587963, and rs7499892), one variant was intergenic (rs12920974) and one variant was exonic (rs34065661) (Table 1). Using PLINK software,^33^ we calculated the LD between the five variants based on the 1000 Genomes reference panel, and found that none are in high LD with each other (Supplementary Table 3).

### Replication of the independent CETP variants

**Figure 3:**
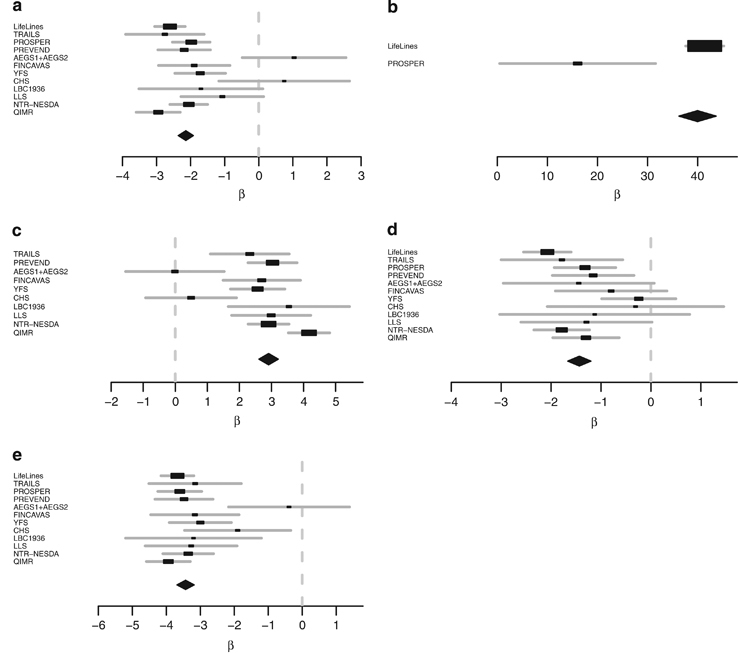
Forest plots of the replication meta-analysis for the five independent variants within the *CETP* region. Only cohorts in which the variants passed QC are included in the forest plot. (**a**) rs12920974 (chromosome 16, position 56,993,025), (**b**) rs34065661 (chromosome 16, position 56,995,935), (**c**) rs5817082 (chromosome 16, position 56,997,349), (**d**) rs4587963 (chromosome 16, position 56,997,369), and (**e**) rs7499892 (chromosome 16, position 57,006,590). *CETP*, cholesteryl ester transfer protein.

The five independent variants within the *CETP* region were selected for replication within the following cohorts: Athero-Express, CHS, FINCAVAS, LBC1936, Lifelines, LLS, NTR-NESDA, PREVEND, PROSPER, QIMR, TRAILS, and YFS. Five variants were replicated at a *P* value of 2.99×10^−34^ (Figure 3 and Table 2).

### Test to explain the previously published results

In each discovery and replication cohort, we tested if the five independent variants explain the associations within the *CETP* region (chromosome 16, 56.99–57.02 Mbp) as reported in the study by Teslovich *et al.*^9^ We tested a total of 38 genome-wide significant (*P* value<5×10^−8^) SNPs within this region identified by Teslovich *et al.*^9^ and conditioned for the five independent variants in all discovery and replication cohorts. All 38 variants were significantly (*P* value corrected for multiple testing<2.85×10^−3^) associated with HDL-C in our joint analyses without adjusting for the 5 independent variants we identified in this work, and 37 (97.37%) were genome-wide significant (*P* value<5×10^−8^) despite the fact that our sample size is about 65% of the study by Teslovich *et al.*^9^ (Table 3). When conditioning on the 5 variants identified in this work, 27 (71.05%) variants remained significant (*P* value<2.85×10^−3^), though the *P* values were markedly reduced (Table 3). This finding suggests that the new variants we identified may explain in part the previously reported association. Remarkably, the *P* value of rs3764261 which was reported as the lead SNP for this *CETP* region by Teslovich *et al.*^9^ was highly reduced from 5.25×10^−509^ to 9.51×10^−25^ while the *β* decreased from 3.179 mg/dl to 0.859 mg/dl. This variant is not in LD with any of the five new variants. Due to the lack of LD, the s.e. of rs3764261 does not change much (s.e._unadj_=0.066, s.e._adj_=0.084), but the effect of rs3764261 does (*β*_unadj_=3.179, *β*_adj_=0.859) and therefore the *χ*^2^ decreases as well, and that results in a higher *P* value. This indicates that a part of the effect of rs3764261 can be explained by the effect of the five new variants.

### Conditional analysis of the independent CETP variants

**Figure 4:**
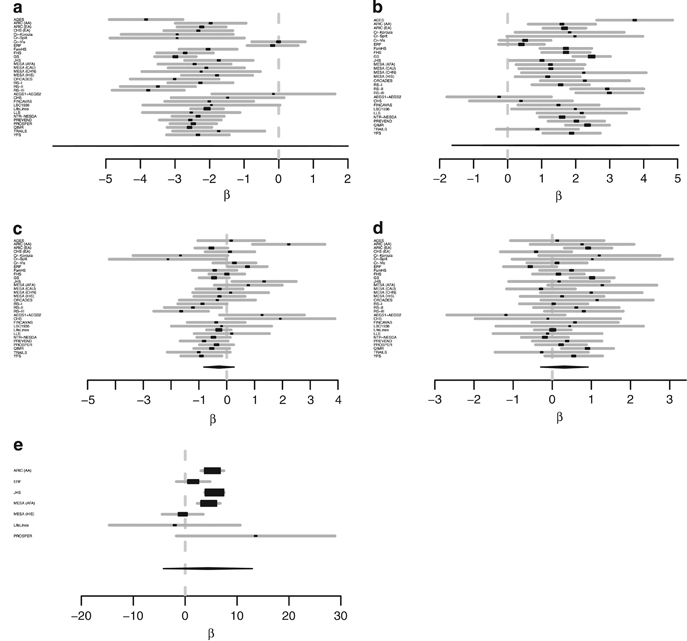
Forest plots of the conditional analysis in the combined discovery and replication cohorts for the five independent variants within the *CETP* region. Only cohorts in which the variants passed quality control (QC) are included in the forest plot. (**a**) rs12920974 (chromosome 16, position 56,993,025), (**b**) rs34065661 (chromosome 16, position 56,995,935), (**c**) rs5817082 (chromosome 16, position 56,997,349), (**d**) rs4587963 (chromosome 16, position 56,997,369), and (**e**) rs7499892 (chromosome 16, position 57,006,590). *CETP*, cholesteryl ester transfer protein.

Next, we performed conditional analysis of the independent variants in both the discovery and replication cohorts. We conditioned on the lead SNP for the *CETP* region as reported by the study by Teslovich *et al.*^9^ (rs3764261, chromosome 16, position 56,993,324 bp), see Table 4 and Figure 4. This analysis showed that three out of the five variants (rs34065661, rs5817082, rs7499892) are independent of rs3764261. For all variants the *P* values and *β*’s decreased, but all *P* values remained significant. The effect of the single variant rs34065661, of the insertion rs5817082, and of the single variant rs7499892 were reduced by 53.20%, 38.48%, and 32.67%, respectively.

### Validation of the insertion within a family

**Figure 5:**
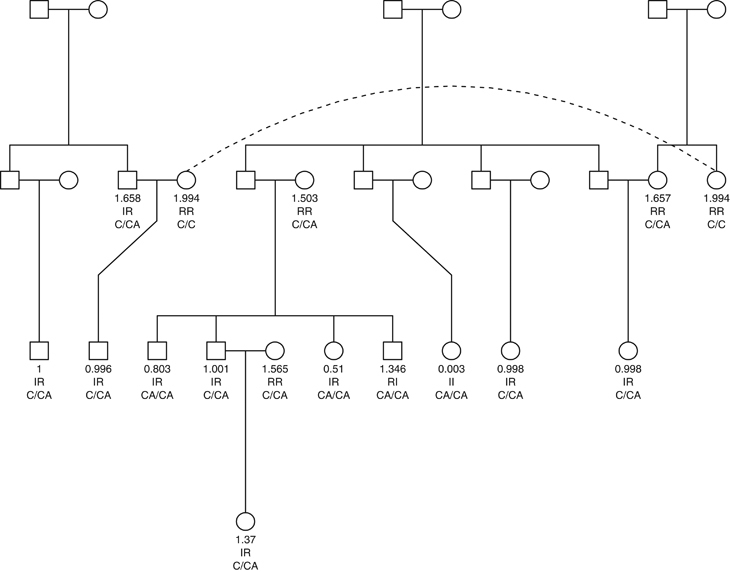
Validation of the insertion (rs5817082) with a large family. The numbers present the dosage for rs5817082 after imputations, second row the best guess result (I is insertion, R is reference) and the third row the genotypes of the insertion from Sanger sequencing.

We selected based on the best guess imputations of the ERF study, a large family of 30 individuals for Sanger sequencing of rs5817082. Using MERLIN^35^ we estimated that the total heritability of HDL-C within this family is 27.47%. DNA was available for 16 individuals. Figure 5 shows the results of the Sanger sequencing for rs5817082 for these 16 individuals within the family. The sequencing of the insertion confirmed the best guess results for 10 individuals (62.5%), of which 7 were heterozygous for the insertion, 1 was homozygous for the insertion, and 2 did not carry the insertion. Three individuals that are homozygous for the insertion, were predicted to be heterozygous by the best guess imputations. Three individuals that are heterozygous for the insertion were not predicted to carry the insertion by the best guess imputations. Furthermore, the Sanger sequencing showed that the insertion segregates with the outcome within this family. The proportion of variance explained by the insertion within this family is 35.50%, while the proportion explained by rs3764261, the lead SNP within the *CETP* region as reported by the study by Teslovich *et al.*^9^ is 14.11%.

## Discussion

We conducted an analysis to fine map the association between *CETP* genetic variants and HDL-C. To this end, a total of 59,432 samples were imputed to the latest version of the 1000 Genomes (version Phase 1 integrated release v3, April 2012, all populations). We identified and replicated five independent variants within the *CETP* region (chromosome 16, 56.99–57.02 Mbp), of which four are SNPs and one is an insertion. We validated the insertion by Sanger sequencing within a large family, as the largest effect on HDL-C comes from this insertion.

The relationship between the *CETP* gene and HDL-C has been known for a long time^9^ and genome-wide association studies have revealed many common and rare variants in this region. Although the associated genetic variants are strongly correlated with HDL-C, the causal variants have not been determined. Our study showed that when using the latest 1000 Genomes reference panel, we have more power to fine map this association. By conditional analysis of the five variants, we were able to reduce the *P* values of the genome-wide significant associations published before by Teslovich *et al.*^9^ Furthermore, conditional analysis showed that three out of the five variants are independent of the lead SNP for the *CETP* region as reported by the study by Teslovich *et al.*^9^ (rs3764261).

Several fine-mapping effort have been previously published^36,37^ and in all those efforts sequencing was used for the fine mapping. In our project we did not use sequencing, but imputations using the 1000 Genomes as a reference panel. This method has been widely used in the past and is much lower in cost. With new reference panels available, we were able to have a revised study of this region. The 1000 Genomes reference panel consists of 30 million variants including a million insertions and deletions. By using this reference panel for imputation, we were able to impute these insertions and deletions in 59,432 samples from various cohorts. This led to the significant association of an insertion within a known region with HDL-C. So far, no association between a structural variation and HDL-C has been found in such a large sample size. Validation of the insertion by Sanger sequencing confirms the correct imputations of this insertion in 62.5% of the individuals, of which seven heterozygous carriers, one homozygous carrier and two did not carry the insertion.

The results of this study showed that by using the 1000 Genomes reference panel, the proportion of the variance explained can be increased and that multiple common variants in the same region may be implicated in a single family of the ERF study. The insertion we identified in this study explains 35.50% of variation in the HDL-C level in a single family of the ERF study; this is in concordance with the results of the whole-genome sequence data.^23^ This is much higher than the proportion of the variance explained (14.11%) in the same family by rs3764261, which was reported before as the lead variant of this region. Fine mapping of various associations may help us to unravel the genetic background of various phenotypes.

Although rs3764261 was identified by Teslovich *et al.*^9^ to be the lead SNP of this region, other variants are used in clinical settings. Three of the classical variants are located in the promoter region of the CETP gene: −1337C/T (rs708272 or Taq1B), −971G/A, and −629C/A (rs1800775) polymorphisms.^38^ Carriers of the B2 allele of the common Taq1B polymorphism exhibit lower plasma CETP levels and higher HDL-C. Furthermore, a recent meta-analysis showed that the B2 allele is associated with a reduced risk for coronary heart disease.^39^ One more classical variant is rs5882A (405I/V), which is located outside the promoter region.^40^ The −1337C/T and −629C/A are in strong LD, however, they are in very low LD (*r*^2^ of 0.442 for rs708272 and 0.461 for rs1800775) with rs3764261, despite the fact that all three variant are within 3,000 bp of each other.

Large HDL-C particle sizes have been associated with exceptional longevity before and with an increased homozygosity for the I405V variant within the *CETP* gene.^1–4^ Many of the studies confirm this relationship, however, all are based on genotyping of the I405V variant. Our study, however, shows that more variants within the *CETP* gene are associated with HDL-C levels in the blood circulation. Therefore we would suggest investigating more variants within the *CETP* gene for its association with longevity and healthy aging.

Some genetic variants identified in our study were published before,^41,42^ but so far no conditional analyses have been performed with these variants. Our study suggests that various *CETP* variants may be relevant for HDL-levels in the blood circulation and that these may have a substantial role in the heritability of HDL-C in specific families.

## Figures and Tables

**Table 1:** The five independent variants after meta-analysis in the discovery cohorts

						*After meta-analysis*				*After GCTA analysis*		
*Marker name*	*Chr*	*Position*	*EA*	*Type*	*Freq*	β^a^	*S.e.*_β_	P *value*	*Freq*_*geno*_	β*_J_* a	*S.e.*_β*j*_	P *value_J_*
rs12920974	16	56,993,025	T	SNP	0.271	−1.748	0.096	1.41E−74	0.281	−1.806	0.139	2.40E−38
rs34065661	16	56,995,935	G	SNP	0.058	7.203	0.560	7.04E−38	0.020	6.782	0.582	2.23E−31
rs5817082	16	56,997,349	CA	INDEL	0.285	−2.869	0.098	8.95E−187	0.305	−4.286	0.172	1.55E−137
rs4587963	16	56,997,369	A	SNP	0.240	−0.972	0.101	5.25E−22	0.261	−2.014	0.165	2.11E−34
rs7499892	16	57,006,590	T	SNP	0.209	−3.384	0.107	2.94E−218	0.245	−2.083	0.150	1.31E−43

Abbreviations: EA, effect allele—the allele for which the effect on HDL-C is estimated; Freq, the frequency of reference allele in the discovery cohorts; Freq_geno_, the frequency of the variant within the reference panel.

a *β* is the effect of the effect allele. *β*_j_ is the effect of the effect allele after joint analysis of all selected variants by GCTA.

**Table 2:** Replication of the 5 independent variants within the *CETP* region

*Marker name*	*Chr*	*Position*	*EA*	*Non effect allele*	*Freq*	β^a^	*S.e.*_β_	P *value*	*Direction of effect per cohort*^b^											
rs12920974	16	56,993,025	T	G	0.288	−2.140	0.112	3.36E−81	−	−	−	−	−	−	−	−	−	−	−	−
rs34065661	16	56,995,935	G	C	0.018	39.958	1.884	8.46E−100	?	?	?	?	+	?	?	?	+	?	?	?
rs5817082	16	56,997,349	CA	C	0.229	−2.911	0.153	1.09E−80	+	−	−	−	?	−	−	−	?	−	−	−
rs4587963	16	56,997,369	A	T	0.325	−1.433	0.117	2.99E−34	−	−	−	−	−	−	−	−	−	−	−	−
rs7499892	16	57,006,590	T	C	0.257	−3.434	0.127	5.64E−160	−	−	−	−	−	−	−	−	−	−	−	−

Abbreviations: *CETP*, cholesteryl ester transfer protein; EA, effect allele—the allele for which the effect on HDL-C is estimated; Freq, the frequency of effect allele.

a *β* is the effect of the effect allele.

b Direction of the effect of the effect allele of the following cohorts: AEGS, CHS (AA), FINCAVAS, LBC1936, Lifelines, LLS, NTR-NESDA, PREVEND, PROSPER, QIMR, TRAILS, and YFS.

The question marks mean that the variant was removed prior to meta-analysis due to a low imputation quality and/or expMAC <10.

**Table 3:** Unadjusted and conditional analysis of the Teslovich variants on the five independent variants in the combined analysis of all discovery and replication cohorts

					*Unadjusted analysis*				*Adjusted analysis*			
*Marker name*	*Chr*	*position*	*EA*	*NEA*	*Freq*	β^a^	*S.e.*_β_	P *value*	*Freq*	β^a^	*S.e.*_β_	P *value*
rs6499861	16	56,991,495	C	G	0.758	1.432	0.090	5.63E−57	0.781	1.083	0.106	1.47E−24
rs6499863	16	56,992,017	A	G	0.251	−1.420	0.093	1.02E−52	0.227	−1.162	0.112	2.59E−25
rs12708967	16	56,993,211	T	C	0.726	2.419	0.087	9.61E−170	0.768	−0.363	0.110	9.99E−04
rs3764261	16	56,993,324	A	C	0.409	3.179	0.066	5.25E−509	0.358	0.859	0.084	9.51E−25
rs12447839	16	56,993,935	T	C	0.665	1.215	0.077	1.87E−56	0.738	0.302	0.111	6.35E−03
rs12447924	16	56,994,192	T	C	0.683	1.218	0.077	8.54E−57	0.737	0.321	0.109	3.15E−03
rs4783961	16	56,994,894	A	G	0.496	1.680	0.064	9.60E−152	0.493	0.732	0.073	6.73E−24
rs4783962	16	56,995,038	T	C	0.318	−1.178	0.081	1.51E−48	0.255	−0.288	0.123	1.97E−02
rs1800775	16	56,995,236	A	C	0.471	2.788	0.064	2.12E−416	0.495	0.547	0.088	4.97E−10
rs711752	16	56,996,211	A	G	0.445	2.782	0.064	3.93E−414	0.435	0.396	0.083	1.56E−06
rs1864163	16	56,997,233	A	G	0.311	−2.991	0.076	1.33E−340	0.238	−0.307	0.115	7.75E−03
rs9929488	16	56,998,572	C	G	0.338	−2.189	0.075	7.55E−189	0.308	0.125	0.092	1.76E−01
rs7203984	16	56,999,258	A	C	0.693	2.903	0.080	2.44E−287	0.737	0.076	0.112	4.95E−01
rs11508026	16	56,999,328	T	C	0.417	2.703	0.065	1.27E−383	0.407	0.326	0.082	7.60E−05
rs820299	16	57,000,284	A	G	0.578	0.892	0.066	8.60E−42	0.595	0.336	0.084	6.07E−05
rs12597002	16	57,002,404	A	C	0.389	−1.228	0.071	2.02E−66	0.307	−0.481	0.103	3.25E−06
rs9926440	16	57,002,663	C	G	0.371	−2.141	0.072	1.18E−196	0.351	0.131	0.085	1.26E−01
rs9939224	16	57,002,732	T	G	0.288	−2.944	0.080	2.72E−300	0.229	0.051	0.109	6.41E−01
rs11076174	16	57,003,146	T	C	0.797	2.388	0.123	1.70E−83	0.825	0.496	0.133	1.99E−04
rs7205804	16	57,004,889	A	G	0.440	2.644	0.063	1.63E−386	0.422	0.291	0.082	3.51E−04
rs1532624	16	57,005,479	A	C	0.420	2.639	0.063	6.82E−386	0.412	0.291	0.082	3.48E−04
rs11076175	16	57,006,378	A	G	0.740	3.326	0.084	5.05E−342	0.815	−0.031	0.127	8.05E−01
rs7499892	16	57,006,590	T	C	0.323	−3.227	0.084	6.95E−323	0.241	−0.197	0.119	9.74E−02
rs289714	16	57,007,451	A	G	0.669	2.624	0.085	6.46E−208	0.708	0.540	0.101	1.01E−07
rs289715	16	57,008,508	A	T	0.256	2.047	0.106	5.38E−83	0.245	0.420	0.106	7.37E−05
rs289717	16	57,009,388	A	G	0.422	−1.357	0.068	1.39E−89	0.401	−0.353	0.077	4.15E−06
rs289719	16	57,009,941	T	C	0.383	1.701	0.070	2.85E−132	0.374	0.461	0.072	1.32E−10
rs4784744	16	57,011,185	A	G	0.396	−1.319	0.066	1.05E−87	0.386	−0.350	0.074	2.37E−06
rs4784745	16	57,014,875	A	G	0.614	1.327	0.068	5.66E−85	0.626	0.314	0.075	3.21E−05
rs5880	16	57,015,091	C	G	0.135	−4.495	0.175	4.42E−146	0.119	−1.331	0.181	1.92E−13
rs5882	16	57,016,092	A	G	0.613	−1.442	0.067	4.19E−102	0.614	−0.410	0.069	2.39E−09
rs9923854	16	57,017,002	T	G	0.802	−1.391	0.115	1.07E−33	0.805	−0.543	0.117	3.28E−06
rs289741	16	57,017,474	A	G	0.631	−1.547	0.068	3.37E−113	0.633	−0.476	0.070	1.02E−11
rs1801706	16	57,017,662	A	G	0.276	1.040	0.091	1.82E−30	0.270	0.493	0.095	1.92E−07
rs289742	16	57,017,762	C	G	0.295	1.811	0.098	1.21E−76	0.285	0.407	0.098	3.40E−05
rs289744	16	57,018,102	T	G	0.641	−1.544	0.069	4.99E−110	0.643	−0.469	0.071	3.33E−11
rs12720917	16	57,019,392	T	C	0.769	−1.474	0.110	1.15E−40	0.775	−0.377	0.109	5.43E−04
rs289745	16	57,019,532	A	C	0.579	0.276	0.081	6.82E−04	0.581	0.204	0.081	1.12E−02

Abbreviations: EA, effect allele for which the effect is estimated; Freq, the frequency of effect allele; NEA, non-effect allele.

a *β* is the effect of effect allele.

**Table 4:** Analysis of the independent variants within the *CETP* region conditioned on the lead SNP for the *CETP* region as reported by the study by Teslovich *et al.*^9^ (rs3764261) in the combined analysis of all discovery and replication cohorts

					*Unadjusted analysis*				*Adjusted analysis*			
*Marker name*	*Chr*	*Position*	*EA*	*NEA*	*Freq*	β^a^	*S.e.*_β_	P *value*	*Freq*	β^a^	*S.e.*_β_	P *value*
rs12920974	16	56,993,025	T	G	0.344	−1.880	0.074	9.91E−143	0.336	−0.278	0.076	2.82E−04
rs34065661	16	56,995,935	C	G	0.854	−9.333	0.520	6.02E−72	0.838	−4.368	0.550	1.94E−15
rs5817082	16	56,997,349	CA	C	0.360	−2.765	0.085	1.49E−231	0.351	−1.701	0.086	2.16E−86
rs4587963	16	56,997,369	A	T	0.351	−1.133	0.077	1.62E−48	0.339	0.309	0.079	8.81E−05
rs7499892	16	57,006,590	T	C	0.317	−3.275	0.082	2.90E−346	0.304	−2.205	0.083	5.14E−156

Abbreviations: *CETP*, cholesteryl ester transfer protein; EA, effect allele for which the effect on HDL-C is estimated; Freq, the frequency of effect allele; SNP, single nucleotide polymorphism.

a *β* is the effect of the effect allele.
