# Supplementary material for: Fine mapping the CETP region reveals a common intronic insertion associated to HDL-C
Source: NPJ Aging Mech Dis. 2015 Nov 12;1:15011–. doi: 10.1038/npjamd.2015.11 (PMC5514988; doi:10.1038/npjamd.2015.11)
Supplement: Supplementary Information [file npjamd201511-s1.doc]

**SUPPLEMENTAL MATERIAL**

# Supplemental: Lifelines cohort study

LifeLines Cohort Study: Behrooz Z Alizadeh (1), Rudolf A de Boer (2), H Marike Boezen (1), Marcel Bruinenberg (3), Lude Franke (4), Pim van der Harst (2), Hans L Hillege (1,2), Melanie M van der Klauw (5), Gerjan Navis (6), Johan Ormel (7), Dirkje S Postma (8), Judith GM Rosmalen (7), Joris P Slaets (9), Harold Snieder (1), Ronald P Stolk (1), Bruce HR Wolffenbuttel (5), Cisca Wijmenga (4)

1. Department of Epidemiology, University of Groningen, University Medical Center Groningen, The Netherlands
2. Department of Cardiology, University of Groningen, University Medical Center Groningen, The Netherlands
3. LifeLines Cohort Study, University of Groningen, University Medical Center Groningen, The Netherlands
4. Department of Genetics, University of Groningen, University Medical Center Groningen, The Netherlands
5. Department of Endocrinology, University of Groningen, University Medical Center Groningen, The Netherlands
6. Department of Internal Medicine, Division of Nephrology, University of Groningen, University Medical Center Groningen, The Netherlands
7. Interdisciplinary Center of Psychopathology of Emotion Regulation (ICPE), Department of Psychiatry, University of Groningen, University Medical Center Groningen, The Netherlands
8. Department of Pulmonology, University of Groningen, University Medical Center Groningen, The Netherlands
9. University Center for Geriatric Medicine, University of Groningen, University Medical Center Groningen, The Netherlands

# Supplemental: CHARGE Lipids Working Group

Tarunveer S. Ahluwalia^1,2,3^, Paul L. Auer^4^, Amy R. Bentley^5^, Lawrence F. Bielak^6^, Joshua C. Bis^7^, Eric Boerwinkle^8^, Ingrid B. Borecki^9^, Jennifer A. Brody^7^, Chris Carlson^10^, Yii-Der Ida Chen^11,12,13^, Jacy R Crosby^8^, L. Adrienne Cupples^14,15^, Gail Davies^16^, Paul S. de Vries^17^, Ian J. Deary^16^, Abbas Dehghan^17^, Ayse Demirkan^17^, Serkalem Demissie^14^, Qing Duan^18^, Gudny Eiriksdottir^19^, Mary F. Feitosa^9^, Myriam Fornage^8,20^, Misa Graff^21^, Vilmundur Gudnason^19,22^, Tamara B. Harris^23^, Caroline Hayward^24^, Albert Hofman^17^, Jennifer E. Huffman^15,24^, Rebecca D. Jackson^25^, Johanna Jakobsdottir^19^, Sharon L. R. Kardia^6^, Sekar Kathiresan^26,27,28,29^, Charles Kooperberg^10^, Leslie A. Lange^18^, Xiaohui Li^11,12^, Yongmei Liu^30^, Ani Manichaikul^31^, Jonathan Marten^24^, Hao Mei^32^, Alanna C. Morrison^8^, Kari North^21^, Chris J. O'Donnell^15^, Jose M. Ordovas^33^, Gina M. Peloso^26,27,28,29^, Patricia A. Peyser^6^, Bruce M. Psaty^34,35,36,37^, Alex P. Reiner^10,35^, Kenneth M. Rice^38^, Stephen S. Rich^31^, Jennifer Robinson^39^, Jerome I. Rotter^11,12^, Albert V. Smith^19,22^, Jennifer A. Smith^6^, Kent D. Taylor^11,12^, Cornelia van Duijn^17^, Elisabeth M. van Leeuwen^17^, James G. Wilson^40^.

1 Faculty of Health and Medical Sciences, University of Copenhagen, Copenhagen, Denmark. 2 Danish Pediatric Asthma Centre, Gentofte Hospital, The Capital Region, Gentofte, Denmark. 3 Diabetic Complications Research, Steno Diabetes Center, Gentofte, Denmark. 4 School of Public Health, University of Wisconsin, Milwaukee, WI, USA. 5 National Human Genome Research Institute, National Institute of Health, Bethesda, MD, USA. 6 Department of Epidemiology, University of Michigan, Ann Arbor, USA. 7 Department of Medicine, University of Washington, Seattle, WA, USA. 8 Human Genetics Center, The University of Texas Health Science Center, Houston, USA. 9 Department of Genetics, Washington University School of Medicine, St. Louis, USA. 10 Division of Public Health Sciences, Fred Hutchinson Cancer Research Center, Seattle, WA, USA. 11 Institute for Translational Genomics and Population Sciences, Los Angeles BioMedical Research Institute at Harbor-UCLA Medical Center, Torrance, CA, USA. 12 Harbor-UCLA Medical Center, Division of Genomic Outcomes, Departments of Pediatrics and Medicine, Torrance, CA, USA. 13 Departments of Pediatrics, Medicine, and Human Genetics, UCLA, Los Angeles, CA, USA. 14 Department of Biostatistics, Boston University School of Public Health, Boston, MA, USA. 15 The Framingham Heart Study, NHLBI Cardiovascular Epidemiology and Human Genomics Branch, Framingham, USA. 16 Centre for Cognitive Ageing and Cognitive Epidemiology, University of Edinburgh, Edinburgh, UK. 17 Department of Epidemiology, Erasmus Medical Center, Rotterdam, The Netherlands. 18 Department of Genetics, University of North Carolina, Chapel Hill, North Carolina, USA. 19 Icelandic Heart Association, Kopavogur, Iceland. 20 Institute of Molecular Medicine, The University of Texas Health Science Center, Houston, USA. 21 Department of Epidemiology, University of North Carolina, Chapel Hill, North Carolina, USA. 22 Faculty of Medicine, University of Iceland, Reykjavik, Iceland. 23 National Institute on Aging, National Institute of Health, Bethesda, MD, USA. 24 MRC Human Genetics Unit, MRC IGMM,University of Edinburgh, Edinburgh, United Kingdom. 25 Department of Medicine, Division of Endocrinology, Diabetes and Metabolism, The Ohio State University, Columbus, OH, USA. 26 Program in Medical and Population Genetics,Broad Institute, Cambridge, MA,USA. 27 Center for Human Genetic Research, Massachusetts General Hospital, Boston, MA,USA. 28 Cardiovascular Research Center, Massachusetts General Hospital, Boston, MA, USA. 29 Program in Medical and Population Genetics, Harvard Medical School, Boston, MA,USA. 30 Department of Epidemiology and Prevention, Wake Forest School of Medicine, Winston Salem, NC, USA. 31 Center for Public Health Genomics, Department of Public Health Sciences, University of Virginia, Charlottesville, VA, USA. 32 Center of Biostatistics and Bioinformatics, University of Mississippi Medical Center, Jackson, MS,USA. 33 Tufts University Jean Mayer USDA Human Nutrition Research Center on Aging Nutrition and Genomics Laboratory, Boston, MA, USA. 34 Cardiovascular Health Research Unit, Department of Medicine, University of Washington, Seattle, WA, USA. 35 Department of Epidemiology, University of Washington, Seattle, WA,USA. 36 Department of Health Services, University of Washington, Seattle, WA, USA. 37 Group Health Research Institute, Group Health Cooperative, Seattle, WA,USA. 38 Department of Biostatistics, University of Washington, Seattle, WA,USA. 39 Department of Epidemiology, University of Iowa, Ames, USA. 40 Department of Physiology and Biophysics, University of Mississippi Medical Center, Jackson, MS, USA.

# Supplemental methods: Cohort descriptions

**Athero-Express Biobank Study (AEGS).** The Athero-Express Biobank Study (http://www.atheroexpress.nl) is an ongoing multicenter longitudinal biobank study that started in 2002, and has been described elsewhere1. In short, patients undergoing carotid (CEA) and femoral endarterectomy at two Dutch tertiary referral centers for vascular surgery are included, and during endarterectomy blood and plaque material is obtained and stored at -80ºC. For this study, we only considered CEA patients.

**Age, Gene/Environment Susceptibility (AGES) Study.** The Age, Gene/Environment Susceptibility (AGES Reykjavik) Study was initiated to examine genetic susceptibility and gene/environment interaction as these contribute to phenotypes common in old age, and represents a continuation of the Reykjavik Study cohort begun in 1967. The study is approved by the Icelandic National Bioethics Committee, (VSN: 00-063) and the Data Protection Authority. The researchers are indebted to the participants for their willingness to participate in the study.

**Atherosclerosis Risk in Communities (ARIC) Study.** The ARIC study has been described in detail previously2. Men and women aged 45-64 years at baseline were recruited from four communities: Forsyth County, North Carolina; Jackson, Mississippi; Minneapolis, Minnesota; and Washington County, Maryland. A total of 15,792 individuals, predominantly White and African American, participated in the baseline examination in 1987-1989, with three triennial follow-up examinations and a fifth exam in 2011-2013. All ARIC individuals provided written, informed consent to participate in research protocols that were approved by the University of North Carolina at Chapel Hill, Chapel Hill, NC institutional review board.

**Cardiovascular Health Study (CHS).** The CHS is a population-based cohort study of risk factors for CHD and stroke in adults greater than or equal to 65 years years conducted across four field centers3. The original predominantly Caucasian cohort of 5,201 persons was recruited in 1989-1990 from random samples of the Medicare eligibility lists; subsequently, an additional predominantly African-American cohort of 687 persons were enrolled for a total sample of 5,888. DNA was extracted from blood samples drawn on all participants at their baseline examination. Genotyping was later performed at the General Clinical Research Center's Phenotyping/Genotyping Laboratory at Cedars-Sinai on participants who consented to genetic testing and had DNA available. European ancestry participants who were free of clinical cardiovascular disease at baseline were genotyped using the Illumina 370CNV BeadChip system and African-Americans were genotyped using the Illumina HumanOmni1-Quad_v1 BeadChip system. For European ancestry participants, additional genotypes from the ITMAT-Broad-CARe (IBC) Illumina iSELECT chip were also used for imputation. CHS participants with available lipid measures for whom genotyping was successful constitute the CHS sample for this study. For this study the European ancestry (EA) samples were included in the discovery phase and the African American (AA) samples were included in the replication phase.

CHS was approved by institutional review committees at each site, the subjects gave informed consent, and those included in the present analysis consented to the use of their genetic information for the study of cardiovascular disease.

**CROATIA-Korcula, CROATIA-Split and CROATIA-Vis.** The CROATIA-Vis study includes unselected adult participants who were recruited in a population-based study during 2003 and 2004 in the villages of Vis and Komiza on the Dalmatian island of Vis. All subjects visited the clinical research centre in the region where they were examined in person and where fasting blood was drawn. Biochemical and physiological measurements were performed, detailed genealogies reconstructed, questionnaire of lifestyle and environmental exposures collected, and blood samples stored for further analyses. CROATIA-Korcula participants were recruited in the same manner from the Dalmatian island of Korcula in 2007 and CROATIA-Split from the mainland Croatian city of Split in 2009-2010. All studies received appropriate ethical approval, and all participants gave informed consent.

**Erasmus Rucphen Family (ERF) Study.** The ERF study has been described in detail previously4. A total of approximately 3,000 participants descend from 22 couples who lived in the Rucphen region in The Netherlands in the 19th century. The 2,755 individuals with genotype data and lipid measurements were included in the current analysis.

**Framingham Heart Study (FHS).** The FHS funded by the National Heart Lung and Blood Institute, is an observational population-based cohort study composed of three generations of Framingham (MA) residents predominately of European descent. The Original cohort (*N* = 5,209) was enrolled in 19485. The children and spouses of the Original cohort comprise the Offspring cohort (*N* = 5,124), which was enrolled in 1971-19756. The Third Generation(*N* = 4,095) consists mostly of the children of the Offspring cohort, and was enrolled in 2002 to 20057. All participants were examined every 4-8 years. DNA for surviving participants was collected in the late 1990s and early 2000s (1995-2005). Cholesterol and genetic data from 3,863 Offspring subjects and 3,508 Third Generation subjects contribute to this paper. All lipids were measured on fasting individuals according to LRC guidelines.

**Family Heart Study (FamHS).** The collection of phenotypes and covariates as well as clinical examination have been previously described for the Family Heart Study8. In brief, the FamHS began in 1992 with the ascertainment of 1,200 families, half randomly sampled and half selected because of an excess of CHD or risk factor abnormalities as compared with age- and sex-specific population rates. The families, with approximately 6,000 subjects, were sampled from four population-based parent studies: the Framingham Heart Study, the Utah Family Tree Study, and two centers for the ARIC study. The participants attended a clinic visit between the years 1994-1996 and a broad range of phenotypes was assessed in the general domains of CHD, atherosclerosis, cardiac and vascular function, inflammation and hemostasis, lipids and lipoproteins, blood pressure, diabetes and insulin resistance, pulmonary function, diet, habitual physical activity, anthropometry, medical history and medication use. Approximately 8 years later, 2,756 EA subjects belonging to the 510 of the largest and most informative pedigrees were invited for a second clinical exam (2002-2004). The most important CHD risk factors were measured again, including lipids, parameters of glucose metabolism, blood pressure, anthropometry, and several biochemical and hematologic markers. In addition, a computed tomography examination provided measures of coronary and aortic calcification, and abdominal and liver fat burden. Medical history and medication use was updated. A total of 3,794 EA subjects, from the first clinic visit, participated in the current study.

**Finnish Cardiovascular Study (FINCAVAS).** The purpose of the Finnish Cardiovascular Study (FINCAVAS) is to construct a risk profile - using genetic, haemodynamic and electrocardiographic (ECG) markers - of individuals at high risk of cardiovascular diseases, events and deaths. All patients scheduled for an exercise stress test at Tampere University Hospital and willing to participate have been recruited between October 2001 and December 2007. The final number of participants is 4,567. In addition to repeated measurement of heart rate and blood pressure, digital high-resolution ECG at 500 Hz was recorded continuously during the entire exercise test, including the resting and recovery phases. About 20% of the patients were examined with coronary angiography. Genetic variations known or suspected to alter cardiovascular function or pathophysiology were analysed to elucidate the effects and interactions of these candidate genes, exercise and commonly used cardiovascular medications.

**Generation Scotland: Scottish Family Health Study (GS:SFHS) .** The GS:SFHS is a collaboration between the Scottish Universities and the NHS, funded by the Chief Scientist Office of the Scottish Government. GS:SFHS is a family-based genetic epidemiology cohort with DNA, other biological samples (serum, urine and cryopreserved whole blood) and socio-demographic and clinical data from ~24,000 volunteers, aged 18-98 years, in ~7,000 family groups. Participants were recruited across Scotland, with some family members from further afield, from 2006-2011. Most (87%) participants were born in Scotland and 96% in the United Kingdom or Ireland. The cohort profile has been published9. GS:SFHS operates under appropriate ethical approvals, and all participants gave written informed consent.

**Jackson Heart Study (JHS).** JHS is a large, population-based observational study evaluating the etiology of cardiovascular diseases and related disorders among African Americans residing in the three counties (Hinds, Madison, and Rankin) that make up the Jackson, Mississippi metropolitan area. Data and biologic materials have been collected from 5,301 participants, including a nested family cohort of 1,498 members of 264 families. The age at enrollment for the unrelated cohort was 35-84 years; the family cohort included related individuals >21 years old. During a baseline examination (2000-2004) and two follow-up examinations (2005-2008 and 2009-2012), participants provided extensive medical and social history, had an array of physical and biochemical measurements and diagnostic procedures, and provided blood for genomic DNA.. The study population is characterized by a high prevalence of diabetes, hypertension, obesity, and related disorders. Annual follow-up interviews and cohort surveillance are ongoing.

**Lothian Birth Cohort 1936 (LBC1936).** The Lothian Birth Cohort 1936 includes surviving participants from the Scottish Mental Survey of 194710^,11^. The cohort consists of 1091 relatively healthy individuals assessed on cognitive and medical traits at about 70 years of age. At baseline the sample of 548 men and 543 women had a mean age 69.6 years (SD = 0.8). They were all Caucasian and almost all lived independently in the Lothian region (Edinburgh city and surrounding area) of Scotland. Genotyping was performed at the Wellcome Trust Clinical Research Facility, Edinburgh. Quality control measures were applied; 1005 participants remained. Among participants with genome-wide data, 910 individuals were available for the present analysis.

**Lifelines.** LifeLines12 is a multi-disciplinary prospective population-based cohort study examining in a unique three-generation design the health and health-related behaviours of 165,000 persons living in the North East region of The Netherlands. It employs a broad range of investigative procedures in assessing the biomedical, socio-demographic, behavioural, physical and psychological factors which contribute to the health and disease of the general population, with a special focus on multimorbidity and complex genetics. This study only includes the individuals of which both genotype and lipid measurements were available.

**Leiden Longevity Study (LLS).** The LLS has been designed to investigate biomarkers of healthy ageing and longevity13 and has been described in detail previously14. It is a family-based study consisting of 1,671 offspring of 421 nonagenarian sibling pairs of Dutch descent, and their 744 partners.

**Multi-Ethnic Study of Atherosclerosis (MESA).** MESA is a study of the characteristics of subclinical cardiovascular disease (disease detected non-invasively before it has produced clinical signs and symptoms) and the risk factors that predict progression to clinically overt cardiovascular disease or progression of the subclinical disease15. MESA researchers study a diverse, population-based sample of 6,814 asymptomatic at baseline men and women aged 45-84. Thirty-eight percent of the recruited participants are white (MESA-CAU), 28% African-American (MESA-AFA), 22% Hispanic (MESA-HIS), and 12% Asian, predominantly of Chinese descent (MESA-CHN). Participants were recruited from six field centers across the United States: Wake Forest University, Columbia University, Johns Hopkins University, University of Minnesota, Northwestern University and University of California - Los Angeles.

**Netherlands Twin Register and Netherlands Study of Depression and Anxiety (NTR-NESDA).** The sample used in the analyses in this study consisted of 5,764 participants of the Netherlands Twin Register (NTR). NTR participants are ascertained because of the presence of twins or triplets in the family and consist of multiples, their parents, siblings and spouses. Twins are born in all strata of society and NTR represents a general sample from the Dutch population. Age ranged between 12 and 89 (median 39), and 62.4% was female16^,17^.

The other 1,816 samples originated from the NESDA cohort with available phenotype data. NESDA is a longitudinal study focusing on the course and consequences of depression and anxiety disorders. Subjects for NESDA were recruited from three sources, namely the general population, mental health organizations and general practices. The vast majority of NESDA subjects is selected for depression and anxiety, but the sample also includes healthy controls without lifetime psychiatric disorders. Age ranged between 18 and 65 in NESDA (median 43), and the proportion of females was 66.1%18. For all analysis, we excluded one monozygotic twin per pair. Additional corrections for family resemblance are analysis specific, and described where appropriate. Lipids were measured from fasting blood samples following standard protocols as described in Willemsen *et al*.17^,19^.

**Orkney Complex Disease studies (ORCADES).** The Orkney Complex Disease Study (ORCADES) is a family-based, cross-sectional study in the isolated Scottish archipelago of Orkney. Genetic diversity in this population is decreased compared to Mainland Scotland, consistent with the high levels of endogamy historically. Fasting blood samples were collected and over 300 health-related phenotypes and environmental exposures were measured in each individual. All participants gave informed consent and the study was approved by Research Ethics Committees in Orkney and Aberdeen20.

**Prevention of Renal and Vascular End stage Disease study (PREVEND).** This is an ongoing prospective study investigating the natural course of increased levels of urinary albumin excretion and its relation to renal and cardiovascular disease. Details of the protocol have been described elsewhere21 (www.prevend.org). Blood samples were obtained in the morning hours. Red blood cell measurements were performed at the 2nd visit (about 4.2 years from baseline).

**Prospective Study of Pravastatin in the Elderly at Risk (PROSPER).** A detailed description of the study has been published elsewhere22^-24^. PROSPER was a prospective multicenter randomized placebo-controlled trial to assess whether treatment with pravastatin diminishes the risk of major vascular events in elderly. Between December 1997 and May 1999, we screened and enrolled subjects in Scotland (Glasgow), Ireland (Cork), and the Netherlands (Leiden). Men and women aged 70-82 years were recruited if they had pre-existing vascular disease or increased risk of such disease because of smoking, hypertension, or diabetes. A total number of 5,804 subjects were randomly assigned to pravastatin or placebo. A large number of prospective tests were performed including Biobank tests and cognitive function measurements. A whole genome wide screening has been performed in the sequential PHASE project with the use of the Illumina 660K beadchip. Of 5,763 subjects DNA was available for genotyping.

**QIMR Twin-Family Studies (QIMR).** Study participants comprised adult twins, their spouses and first-degree relatives who volunteered for studies on risk factors or biomarkers for physical or psychiatric conditions. These studies were approved by The Queensland Institute of Medical Research Human Research Ethics Committee. Subjects were not required to fast before blood collection. Lipid measurements were made using Roche methods on Hitachi 917 or Modular p analysers.

**Rotterdam Study cohort I (RS-I).** The Rotterdam Study is an ongoing prospective population-based cohort study, focused on chronic disabling conditions of the elderly. The study comprises an outbred ethnically homogenous population of Dutch Caucasian origin. The rationale of the study has been described in detail elsewhere25. In summary, 7,983 men and women aged 55 years or older, living in Ommoord, a suburb of Rotterdam, the Netherlands, were invited to participate in the first phase. Fasting blood samples were taken during the participant's third visit to the research center.

**Rotterdam Study cohort II (RS-II).** The Rotterdam Study cohort II prospective population-based cohort study comprises 3,011 residents aged 55 years and older from the same district of Rotterdam. The rationale and study designs of this cohort is similar to that of the RS-I25. The baseline measurements, including the fasting HDL measurements, took place during the first visit.

**Rotterdam Study cohort III (RS-III).** The Rotterdam Study cohort III prospective population-based cohort study comprised 3,932 residents aged 45 years and older from the same district of Rotterdam. The rationale and study designs of this cohort is similar to that of the RS-I25. The baseline measurements, including the fasting HDL measurements, took place during the first visit.

**TRacking Adolescents' Individual Lives Survey (TRAILS).** TRAILS is a prospective cohort study of Dutch adolescents with bi- or triennial measurements from age 11 to up until adulthood, which consists of a general population and a clinical cohort (for a cohort profile see Huisman *et al*., 200826. In the population cohort, five assessment waves have been completed to date, which ran from March 2001 to July 2002 (T1), September 2003 to December 2004 (T2), September 2005 to August 2007 (T3), October 2008 to September 2010 (T4), and January 2012 to December 2013 (T5). Data for the present study were collected during the third assessment wave. At T1, 2230 (pre)adolescents were enrolled in the study (response rate 76.0%, mean age 11.09, SD 0.55, 50.8% girls27, of whom 81.4% (N = 1816, mean age 16.27, SD 0.73, 52.3% girls) participated at T3. We obtained a blood sample after >8 h of fasting for the measurement of triglycerides, total cholesterol, and HDL cholesterol (Roche Diagnostics). In this study only the HDL cholesterol measurements were used.

**Young Finns Styudy (YFS).** The YFS is a population-based follow up-study started in 1980. The main aim of the YFS is to determine the contribution made by childhood lifestyle, biological and psychological measures to the risk of cardiovascular diseases in adulthood. In 1980, over 3,500 children and adolescents all around Finland participated in the baseline study. The follow-up studies have been conducted mainly with 3-year intervals. The latest 30-year follow-up study was conducted in 2010-11 (ages 33-49 years) with 2,063 participants. The study was approved by the local ethics committees (University Hospitals of Helsinki, Turku, Tampere, Kuopio and Oulu) and was conducted following the guidelines of the Declaration of Helsinki. All participants gave their written informed consent.

# Supplemental acknowledgements

The Athero-Express Biobank Study acknowledges the support from the Netherlands CardioVascular Research Initiative from the Dutch Heart Foundation, Dutch Federation of University Medical Centres, the Netherlands Organisation for Health Research and Development and the Royal Netherlands Academy of Sciences. We would like to thank Freerk van Dijk and Morris Swertz and acknowledge them graciously for imputing our datasets using the “GoNL Impute2” pipeline.

The authors thank the staff and participants of the ARIC study for their important contributions.

The CROATIA cohorts would like to acknowledge the invaluable contributions of the recruitment teams in Vis, Korcula and Split (including those from the Institute of Anthropological Research in Zagreb and the Croatian Centre for Global Health at the University of Split), the administrative teams in Croatia and Edinburgh and the people of Vis, Korcula and Split. SNP genotyping was performed at the Wellcome Trust Clinical Research Facility in Edinburgh for CROATIA-Vis, by Helmholtz Zentrum München, GmbH, Neuherberg, Germany for CROATIA-Korcula and by AROS Applied Biotechnology, Aarhus, Denmark for CROATIA-Split.

We are grateful to all study participants and their relatives, general practitioners and neurologists for their contributions to the ERF study and to P. Veraart for her help in genealogy, J. Vergeer for the supervision of the laboratory work and P. Snijders for his help in data collection.

This research was conducted in part using data and resources from the Framingham Heart Study of the National Heart Lung and Blood Institute of the National Institutes of Health and Boston University School of Medicine. The analyses reflect intellectual input and resource development from the Framingham Heart Study investigators participating in the SNP Health Association Resource (SHARe) project. This work was partially supported by the National Heart, Lung and Blood Institute's

Framingham Heart Study (Contract No. N01-HC-25195) and its contract with Affymetrix, Inc for genotyping services (Contract No. N02-HL-6-4278). A portion of this research utilized the Linux Cluster for Genetic Analysis (LinGA-II) funded by the Robert Dawson Evans Endowment of the Department of Medicine at Boston University School of Medicine and Boston Medical Center.

On behalf of FINCAVAS, the authors thank the staff of the Department of Clinical Physiology for collecting the exercise test data.

On behalf of LBC1936, we thank the cohort participants and team members who contributed to these studies. Phenotype collection was supported by Age UK (The Disconnected Mind project). Genotyping was funded by the Biotechnology and Biological Sciences Research Council (BBSRC). The work was undertaken by The University of Edinburgh Centre for Cognitive Ageing and Cognitive Epidemiology, part of the cross council Lifelong Health and Wellbeing Initiative (MR/K026992/1). Funding from the BBSRC and Medical Research Council (MRC) is gratefully acknowledged.

On behalf of GS:SFHS we would like to acknowledge the invaluable contributions of the families who took part in the GS:SFHS, the general practitioners and Scottish School of Primary Care for their help in recruiting them, and the whole Generation Scotland team, which includes academic researchers, IT staff, laboratory technicians, statisticians and research managers. SNP genotyping was performed at the Wellcome Trust Clinical Research Facility in Edinburgh.

On behalf of MESA, the authors thank the participants of the MESA study, the Coordinating Center, MESA investigators, and study staff for their valuable contributions. A full list of participating MESA investigators and institutions can be found at http://www.mesa-nhlbi.org.

On behalf of ORCADES, we would like to acknowledge the invaluable contributions of the research nurses in Orkney, the administrative team in Edinburgh and the people of Orkney.

On behalf of the Rotterdam Study, we thank Pascal Arp, Mila Jhamai, Marijn Verkerk, Lizbeth Herrera and Marjolein Peters for their help in creating the GWAS database, and Karol Estrada and Maksim V. Struchalin for their support in creation and analysis of imputed data. The authors are grateful to the study participants, the staff from the Rotterdam Study and the participating general practitioners and pharmacists.

On behalf of YFS, the expert technical assistance in the statistical analyses by Ville Aalto and Irina Lisinen is gratefully acknowledged.

# Supplemental funding sources

This research was financially supported by BBMRI-NL, a Research Infrastructure financed by the Dutch government (NWO 184.021.007). Statistical analyses were carried out on the Genetic Cluster Computer (http://www.geneticcluster.org) which is financially supported by the Netherlands Scientific Organization (NWO 480-05-003 PI: Posthuma) along with a supplement from the Dutch Brain Foundation and the VU University Amsterdam.

Sander W. van der Laan is funded through grants from the Netherlands CardioVascular Research Initiative (“GENIUS”, CVON2011-19), the Interuniversity Cardiology Institute of the Netherlands (ICIN, 09.001) and the FP7 EU project CVgenes@target (HEALTH-F2-2013-601456). Cavadis B.V. (Rotterdam, the Netherlands, www.cavadis.com) financed the genotyping of AEGS1.

The ARIC Study is carried out as a collaborative study supported by National Heart, Lung, and Blood Institute (NHLBI) contracts (HHSN268201100005C, HHSN268201100006C, HHSN268201100007C, HHSN268201100008C, HHSN268201100009C, HHSN268201100010C, HHSN268201100011C, and HHSN268201100012C), R01HL087641, R01HL59367 and R01HL086694; National Human Genome Research Institute contract U01HG004402; and National Institutes of Health contract HHSN268200625226C. Infrastructure of the ARIC study was partly supported by Grant Number UL1RR025005, a component of the National Institutes of Health and NIH Roadmap for Medical Research. Aniko Sabo was funded by NIH (grant number:  U54 HG003273).

This CHS research was supported by NHLBI contracts HHSN268201200036C, HHSN268200800007C, N01HC55222, N01HC85079, N01HC85080, N01HC85081, N01HC85082, N01HC85083, N01HC85086, HHSN268200960009C; and NHLBI grants U01HL080295, R01HL087652, R01HL105756, R01HL103612, and R01HL120393 with additional contribution from the National Institute of Neurological Disorders and Stroke (NINDS). Additional support was provided through R01AG023629 from the National Institute on Aging (NIA). A full list of principal CHS investigators and institutions can be found at CHS-NHLBI.org. The provision of genotyping data was supported in part by the National Center for Advancing Translational Sciences, CTSI grant UL1TR000124, and the National Institute of Diabetes and Digestive and Kidney Disease Diabetes Research Center (DRC) grant DK063491 to the Southern California Diabetes Endocrinology Research Center.

The content is solely the responsibility of the authors and does not necessarily represent the official views of the National Institutes of Health.

The CROATIA studies were supported through the grants from the Medical Research Council UK and Ministry of Science, Education and Sport of the Republic of Croatia (number 108-1080315-0302) and the European Union framework program 6 EUROSPAN project (contract no. LSHG-CT-2006-018947).

The ERF study as a part of EUROSPAN (European Special Populations Research Network) was supported by European Commission FP6 STRP grant number 018947 (LSHG-CT-2006-01947) and also received funding from the European Community's Seventh Framework Programme (FP7/2007-2013)/grant agreement HEALTH-F4-2007-201413 by the European Commission under the programme "Quality of Life and Management of the Living Resources" of 5th Framework Programme (no. QLG2-CT-2002-01254). The ERF study was further supported by ENGAGE consortium and CMSB. High-throughput analysis of the ERF data was supported by joint grant from Netherlands Organisation for Scientific Research and the Russian Foundation for Basic Research (NWO-RFBR 047.017.043). Exome sequencing in ERF was supported by the ZonMw grant (project 91111025).

The FamHS is funded by a NHLBI grant 5R01HL08770003, and NIDDK grants 5R01DK06833603 and 5R01DK07568102.

The work of FINCAVAS was supported by the Competitive Research Funding of the Tampere University Hospital (Grant 9M048 and 9N035), the Finnish Cultural Foundation, the Finnish Foundation for Cardiovascular Research, the Emil Aaltonen Foundation, Finland, and the Tampere Tuberculosis Foundation.

LBC1936 is funded by Age UK, the Biotechnology and Biological Sciences Research Council (BBSRC), the Medical Research Council (MRC) and the Lifelong Health and Wellbeing Initiative (MR/K026992/1).

GS:SFHS is funded by the Scottish Executive Health Department, Chief Scientist Office, grant number CZD/16/6. SNP genotyping was funded by the Medical Research Council UK.

The LifeLines Cohort Study, and generation and management of GWAS genotype data for the LifeLines Cohort Study is supported by the Netherlands Organization of Scientific Research NWO (grant 175.010.2007.006), the Economic Structure Enhancing Fund (FES) of the Dutch government, the Ministry of Economic Affairs, the Ministry of Education, Culture and Science, the Ministry for Health, Welfare and Sports, the Northern Netherlands Collaboration of Provinces (SNN), the Province of Groningen, University Medical Center Groningen, the University of Groningen, Dutch Kidney Foundation and Dutch Diabetes Research Foundation.

The Leiden Longevity Study has received funding from the European Union's Seventh Framework Programme (FP7/2007-2011) under grant agreement n° 259679. This study was supported by a grant from the Innovation-Oriented Research Program on Genomics (SenterNovem IGE05007), the Centre for Medical Systems Biology, and the Netherlands Consortium for Healthy Ageing (grant 050-060-810), all in the framework of the Netherlands Genomics Initiative, Netherlands Organization for Scientific Research (NWO), UnileverColworth and by BBMRI-NL, a Research Infrastructure financed by the Dutch government (NWO 184.021.007).

MESA and the MESA SHARe project are conducted and supported by contracts N01-HC-95159, N01-HC-95160, N01-HC-95161, N01-HC-95162, N01-HC-95163, N01-HC-95164, N01-HC-95165, N01-HC-95166, N01-HC-95167, N01-HC-95168, N01-HC-95169 and RR-024156 from the National Heart, Lung, and Blood Institute (NHLBI) and RR024156 and ES09089. MESA Air is conducted and supported by the United States Environmental Protection Agency (EPA) in collaboration with MESA Air investigators, with support provided by grant RD83169701. Funding for MESA SHARe genotyping was provided by NHLBI Contract N02-HL-6-4278, the National Center for Advancing Translational Sciences, CTSI grant UL1TR000124, and the National Institute of Diabetes and Digestive and Kidney Disease Diabetes Research Center (DRC) grant DK063491 to the Southern California Diabetes Endocrinology Research Center.

Funding of the Netherland Twin Register (NTR) and Netherlands Study of Depression and Anxiety (NESDA) was obtained from the Netherlands Organization for Scientific Research (NWO) and MagW/ZonMW grants Middelgroot-911-09-032, Spinozapremie 56-464-14192, Geestkracht program of the Netherlands Organization for Health Research and Development (Zon-MW, grant number 10-000-1002), Center for Medical Systems Biology (CSMB, NWO Genomics), NBIC/BioAssist/RK(2008.024), Biobanking and Biomolecular Resources Research Infrastructure (BBMRI-NL, 184.021.007), VU University’s Institute for Health and Care Research (EMGO+) and Neuroscience Campus Amsterdam (NCA); the European Science Foundation (ESF, EU/QLRT-2001-01254), the European Community's Seventh Framework Program (FP7/2007-2013), ENGAGE (HEALTH-F4-2007-201413); the European Science Council (ERC Advanced, 230374); the European Research Council (ERC-284167). Part of the genotyping and analyses were funded by the Genetic Association Information Network (GAIN) of the Foundation for the National Institutes of Health, Rutgers University Cell and DNA Repository (NIMH U24 MH068457-06), the Avera Institute, Sioux Falls, South Dakota (USA) and the National Institutes of Health (NIH R01 HD042157-01A1, MH081802, Grand Opportunity grants 1RC2 MH089951 and 1RC2 MH089995).

The PROSPER study was supported by an investigator initiated grant obtained from Bristol-Myers Squibb. Prof. Dr. J. W. Jukema is an Established Clinical Investigator of the Netherlands Heart Foundation (grant 2001 D 032). Support for genotyping was provided by the seventh framework program of the European commission (grant 223004) and by the Netherlands Genomics Initiative (Netherlands Consortium for Healthy Aging grant 050-060-810).

ORCADES was supported by the Chief Scientist Office of the Scottish Government, the Royal Society, the UK Medical Research Council Human Genetics Unit, Arthritis Research UK and the European Union framework program 6 EUROSPAN project (contract no. LSHG-CT-2006-018947). DNA extractions were performed at the Wellcome Trust Clinical Research Facility in Edinburgh.

On behalf of QIMR Twin-Family Studies, we acknowledge funding from the Australian National Health and Medical Research Council (NHMRC grants 241944, 389875, 389891,389892, 389938, 442915, 442981, 496739 and 552485), US National Institutes of Health (AA07535, AA10248 and AA014041).

The generation and management of GWAS genotype data for the Rotterdam Study is supported by the Netherlands Organisation of Scientific Research NWO Investments (nr. 175.010.2005.011, 911-03-012). This study is funded by the Research Institute for Diseases in the Elderly (014-93-015; RIDE2), the Netherlands Genomics Initiative (NGI)/Netherlands Organisation for Scientific Research (NWO) project nr. 050-060-810. The Rotterdam Study is funded by Erasmus Medical Center and Erasmus University, Rotterdam, Netherlands Organization for the Health Research and Development (ZonMw), the Research Institute for Diseases in the Elderly (RIDE), the Ministry of Education, Culture and Science, the Ministry for Health, Welfare and Sports, the European Commission (DG XII), and the Municipality of Rotterdam.

Abbas Dehghan is supported by Netherlands Organisation for Scientific Research (NWO) grant (veni, 916.12.154) and the EUR Fellowship.

TRAILS (TRacking Adolescents’ Individual Lives Survey) is a collaborative project involving various departments of the University Medical Center and University of Groningen, the Erasmus University Medical Center Rotterdam, the University of Utrecht, the Radboud Medical Center Nijmegen, and the Parnassia Bavo group, all in the Netherlands. TRAILS has been financially supported by grants from the Netherlands Organization for Scientific Research NWO (Medical Research Council program grant GB-MW 940-38-011; ZonMW Brainpower grant 100-001-004; ZonMw Risk Behavior and Dependence grant 60-60600-97-118; ZonMw Culture and Health grant 261-98-710; Social Sciences Council medium-sized investment grants GB-MaGW 480-01-006 and GB-MaGW 480-07-001; Social Sciences Council project grants GB-MaGW 452-04-314 and GB-MaGW 452-06-004; NWO large-sized investment grant 175.010.2003.005; NWO Longitudinal Survey and Panel Funding 481-08-013); the Dutch Ministry of Justice (WODC), the European Science Foundation (EuroSTRESS project FP-006), Biobanking and Biomolecular Resources Research Infrastructure BBMRI-NL (CP 32), the participating universities, and Accare Center for Child and Adolescent Psychiatry. We are grateful to all adolescents, their parents and teachers who participated in this research and to everyone who worked on this project and made it possible. Statistical analyses were carried out on the Genetic Cluster Computer (http://www.geneticcluster.org), which is financially supported by the Netherlands Scientific Organization (NWO 480-05-003) along with a supplement from the Dutch Brain Foundation.

The Young Finns Study has been financially supported by the Academy of Finland: grants 134309 (Eye), 126925, 121584, 124282, 129378 (Salve), 117787 (Gendi), and 41071 (Skidi), the Social Insurance Institution of Finland, Kuopio, Tampere and Turku University Hospital Medical Funds (grant 9M048 and 9N035 for TeLeht), Juho Vainio Foundation, Paavo Nurmi Foundation, Finnish Foundation of Cardiovascular Research and Finnish Cultural Foundation, Tampere Tuberculosis Foundation and Emil Aaltonen Foundation (T.L).

# Supplemental references

1. Verhoeven BAN, Velema E, Schoneveld AH, de Vries JPPM, de Bruin P, Seldenrijk CA, et al. Athero-express: differential atherosclerotic plaque expression of mRNA and protein in relation to cardiovascular events and patient characteristics. Rationale and design. Eur J Epidemiol. 2004;19(12):1127–1133.

2. The Atherosclerosis Risk in Communities (ARIC) Study: design and objectives. The ARIC investigators. Am J Epidemiol. 1989 Apr;129(4):687–702.

3. Fried LP, Borhani NO, Enright P, Furberg CD, Gardin JM, Kronmal RA, et al. The Cardiovascular Health Study: design and rationale. Ann Epidemiol. 1991 Feb;1(3):263–276.

4. Pardo LM, MacKay I, Oostra B, van Duijn CM, Aulchenko YS. The effect of genetic drift in a young genetically isolated population. Ann Hum Genet. 2005 May;69(Pt 3):288–295. Available from: http://­dx.doi.org/­10.1046/­j.1529-8817.2005.00162.x.

5. Dawber TR, Kannel WB, Lyell LP. An approach to longitudinal studies in a community: the Framingham Study. Ann N Y Acad Sci. 1963 May;107:539–556.

6. Feinleib M, Kannel WB, Garrison RJ, McNamara PM, Castelli WP. The Framingham Offspring Study. Design and preliminary data. Prev Med. 1975 Dec;4(4):518–525.

7. Splansky GL, Corey D, Yang Q, Atwood LD, Cupples LA, Benjamin EJ, et al. The Third Generation Cohort of the National Heart, Lung, and Blood Institute’s Framingham Heart Study: design, recruitment, and initial examination. Am J Epidemiol. 2007 Jun;165(11):1328–1335. Available from: http://­dx.doi.org/­10.1093/­aje/­kwm021.

8. Higgins M, Province M, Heiss G, Eckfeldt J, Ellison RC, Folsom AR, et al. NHLBI Family Heart Study: objectives and design. Am J Epidemiol. 1996 Jun;143(12):1219–1228.

9. Smith BH, Campbell A, Linksted P, Fitzpatrick B, Jackson C, Kerr SM, et al. Cohort Profile: Generation Scotland: Scottish Family Health Study (GS:SFHS). The study, its participants and their potential for genetic research on health and illness. Int J Epidemiol. 2013 Jun;42(3):689–700. Available from: http://­dx.doi.org/­10.1093/­ije/­dys084.

10. Deary IJ, Gow AJ, Taylor MD, Corley J, Brett C, Wilson V, et al. The Lothian Birth Cohort 1936: a study to examine influences on cognitive ageing from age 11 to age 70 and beyond. BMC Geriatr. 2007;7:28. Available from: http://­dx.doi.org/­10.1186/­1471-2318-7-28.

11. Deary IJ, Gow AJ, Pattie A, Starr JM. Cohort profile: the Lothian Birth Cohorts of 1921 and 1936. Int J Epidemiol. 2012 Dec;41(6):1576–1584. Available from: http://­dx.doi.org/­10.1093/­ije/­dyr197.

12. Stolk RP, Rosmalen JGM, Postma DS, de Boer RA, Navis G, Slaets JPJ, et al. Universal risk factors for multifactorial diseases: LifeLines: a three-generation population-based study. Eur J Epidemiol. 2008;23(1):67–74. Available from: http://­dx.doi.org/­10.1007/­s10654-007-9204-4.

13. Westendorp RGJ, van Heemst D, Rozing MP, Frölich M, Mooijaart SP, Blauw GJ, et al. Nonagenarian siblings and their offspring display lower risk of mortality and morbidity than sporadic nonagenarians: The Leiden Longevity Study. J Am Geriatr Soc. 2009 Sep;57(9):1634–1637.

14. Schoenmaker M, de Craen AJM, de Meijer PHEM, Beekman M, Blauw GJ, Slagboom PE, et al. Evidence of genetic enrichment for exceptional survival using a family approach: the Leiden Longevity Study. Eur J Hum Genet. 2006 Jan;14(1):79–84. Available from: http://­dx.doi.org/­10.1038/­sj.ejhg.5201508.

15. Bild DE, Bluemke DA, Burke GL, Detrano R, Diez Roux AV, Folsom AR, et al. Multi-ethnic study of atherosclerosis: objectives and design. Am J Epidemiol. 2002 Nov;156(9):871–881.

16. Boomsma DI, de Geus EJC, Vink JM, Stubbe JH, Distel MA, Hottenga JJ, et al. Netherlands Twin Register: from twins to twin families. Twin Res Hum Genet. 2006 Dec;9(6):849–857. Available from: http://­dx.doi.org/­10.1375/­183242706779462426.

17. Willemsen G, de Geus EJC, Bartels M, van Beijsterveldt CEMT, Brooks AI, van Burk GFE, et al. The Netherlands Twin Register biobank: a resource for genetic epidemiological studies. Twin Res Hum Genet. 2010 Jun;13(3):231–245. Available from: http://­dx.doi.org/­10.1375/­twin.13.3.231.

18. Penninx BWJH, Beekman ATF, Smit JH, Zitman FG, Nolen WA, Spinhoven P, et al. The Netherlands Study of Depression and Anxiety (NESDA): rationale, objectives and methods. Int J Methods Psychiatr Res. 2008;17(3):121–140.

19. van Reedt Dortland AKB, Giltay EJ, van Veen T, van Pelt J, Zitman FG, Penninx BWJH. Associations between serum lipids and major depressive disorder: results from the Netherlands Study of Depression and Anxiety (NESDA). J Clin Psychiatry. 2010 Jun;71(6):729–736. Available from: http://­dx.doi.org/­10.4088/­JCP.08m04865blu.

20. McQuillan R, Leutenegger AL, Abdel-Rahman R, Franklin CS, Pericic M, Barac-Lauc L, et al. Runs of homozygosity in European populations. Am J Hum Genet. 2008 Sep;83(3):359–372. Available from: http://­dx.doi.org/­10.1016/­j.ajhg.2008.08.007.

21. Hillege HL, Janssen WM, Bak AA, Diercks GF, Grobbee DE, Crijns HJ, et al. Microalbuminuria is common, also in a nondiabetic, nonhypertensive population, and an independent indicator of cardiovascular risk factors and cardiovascular morbidity. J Intern Med. 2001 Jun;249(6):519–526.

22. Shepherd J, Blauw GJ, Murphy MB, Cobbe SM, Bollen EL, Buckley BM, et al. The design of a prospective study of Pravastatin in the Elderly at Risk (PROSPER). PROSPER Study Group. PROspective Study of Pravastatin in the Elderly at Risk. Am J Cardiol. 1999 Nov;84(10):1192–1197.

23. Shepherd J, Blauw GJ, Murphy MB, Bollen ELEM, Buckley BM, Cobbe SM, et al. Pravastatin in elderly individuals at risk of vascular disease (PROSPER): a randomised controlled trial. Lancet. 2002 Nov;360(9346):1623–1630.

24. Trompet S, de Craen AJM, Postmus I, Ford I, Sattar N, Caslake M, et al. Replication of LDL GWAs hits in PROSPER/PHASE as validation for future (pharmaco)genetic analyses. BMC Med Genet. 2011;12:131.

25. Hofman A, Darwish Murad S, van Duijn CM, Franco OH, Goedegebure A, Ikram MA, et al. The Rotterdam Study: 2014 objectives and design update. Eur J Epidemiol. 2013 Nov;28(11):889–926. Available from: http://­dx.doi.org/­10.1007/­s10654-013-9866-z.

26. Huisman M, Oldehinkel AJ, de Winter A, Minderaa RB, de Bildt A, Huizink AC, et al. Cohort profile: the Dutch ’TRacking Adolescents’ Individual Lives’ Survey’; TRAILS. Int J Epidemiol. 2008 Dec;37(6):1227–1235. Available from: http://­dx.doi.org/­10.1093/­ije/­dym273.

27. de Winter AF, Oldehinkel AJ, Veenstra R, Brunnekreef JA, Verhulst FC, Ormel J. Evaluation of non-response bias in mental health determinants and outcomes in a large sample of pre-adolescents. Eur J Epidemiol. 2005;20(2):173–181.

28. Aulchenko YS, Ripke S, Isaacs A, van Duijn CM. GenABEL: an R library for genome-wide association analysis. Bioinformatics. 2007 May;23(10):1294–1296. Available from: http://­dx.doi.org/­10.1093/­bioinformatics/­btm108.

29. Svishcheva GR, Axenovich TI, Belonogova NM, van Duijn CM, Aulchenko YS. Rapid variance components-based method for whole-genome association analysis. Nat Genet. 2012 Oct;44(10):1166–1170. Available from: http://­dx.doi.org/­10.1038/­ng.2410.

# Supplemental tables

**Supplemental table 1a.** Baseline characteristics for the discovery cohorts.

Abbreviations: PC (principal component), LLM (lipid lowering medication)

| **Cohort** | ***N***  **(% male)** | **Mean age (SD), in years** | **Mean HDL-C (SD), in mg/dL** | **Fasted** | **Ancestry** | **Cohort specific covariates** | **# individuals using LLM** |
| --- | --- | --- | --- | --- | --- | --- | --- |
| AGES | 3219 (42.00) | 76.41 (5.46) | 61.18 (17.32) | yes | European | PC1-2 | 729 individuals |
| ARIC (AA) | 2733 (37.58) | 53.40 (5.77) | 55.00 (17.35) | not all* | European | center | na** |
| ARIC (EA) | 9471 (46.95) | 54.32 (5.69) | 50.55 (16.69) | not all* | African American | center | na** |
| CHS (EA) | 3188 (39%) | 72.34 (5.39) | 55.3 (15.92) | yes | European | study site | 45 individuals |
| CROATIA-KORCULA | 894 (36.1) | 56.26 (13.98) | 56.48 (13.31) | yes | European | none | 32 individuals |
| CROATIA-SPLIT | 490 (42.0) | 49.04 (14.57) | 53.45 (12.94) | yes | European | none | 1 individual |
| CROATIA-VIS | 947 (42.2) | 56.16 (15.54) | 42.80 (6.07) | yes | European | none | 25 individuals |
| ERF | 2739 (44.61) | 48.96 (14.38) | 49.18 (14.08) | yes | European | family relationships (grammar-gamma from GenABEL version 1.7.628^,29^) | 351 individuals |
| FHS | 3481 (47.2) | 37.86 (9.70) | 52.79 (15.48) | yes | European | Sex, age, age^2^  , PC1-PC10 (population structure PCs), and cohort-generation indicator | 305 individuals |
| FamHS | 3794 | 52.13 (13.63) | 49.74 (14.83) | yes | European | field centers, Illumina chips (550k, 610K, and 1M), 5 PCs | 360 individuals |
| Generation Scotland | 9556 (41.6) | 52.31 (13.56) | 57.18 (16.34) | majority | European | none | 1384 individuals |
| JHS | 1985 (39.24) | 49.69 (12.03) | 50.21 (14.14) | yes | African American | First 10 PCs of ancestry | 167 individuals |
| MESA (AFA) | 1758 (46.13) | 61.69 (10.04) | 52.08 (14.42) | yes | African | study site, first PC of ancestry | 300 individuals |
| MESA (CAU) | 2492 (48.27) | 62.82 (10.17) | 52.14 ( 15.11) | yes | European | study site, first two PCs of ancestry | 473 individuals |
| MESA (CHN) | 703 (49.64) | 62.54 (10.33) | 48.94 (11.83) | yes | Chinese | study site, first PC of ancestry | 97 individuals |
| MESA (HIS) | 1407 (48.90) | 61.22 (10.13) | 47.03 (12.00) | yes | Hispanic | study site, first three PCs of ancestry | 197 individuals |
| ORCADES | 1991 (39.68) | 53.67 (15.37) | 57.22 (15.54) | yes | European | array, PC1, PC2, PC3, and family relationships (polygenic from GenABEL in combination with palinear from ProbABEL) | 246 individuals |
| RS-I | 3410 (57.07) | 65.94 (7.04) | 53.62 (15.30) | yes | European | none | 416 individuals |
| RS-II | 2137 (45.53) | 64.77 (7.98) | 52.98 (14.27) | yes | European | none | 407 individuals |
| RS-III | 3037 (43.66) | 57.10 (6.85) | 52.98 (14.27) | yes | European | none | 407 individuals |

* HDL values were not adjusted

** data is collected before 1994

**Supplemental table 1b.** Baseline characteristics for the replication cohorts.

| **Cohort** | ***N***  **(% male)** | **Mean age (SD), in years** | **Mean HDL-C (SD), in mg/dL** | **Fasted** | **Ancestry** | **Cohort specific covariates** | **# individuals using LLM** |
| --- | --- | --- | --- | --- | --- | --- | --- |
| Athero-Express | 917 (67.94) | 68.32 (9.21) | 45.12 (14.38) | yes | European | Year of surgery, PC1-10, genotyping-chip | 691 individuals |
| CHS (AA) | 785 (37) | 72.82 (5.63) | 58.04 (15.5) | yes | African-American | study site | 33 individuals |
| FINCAVAS | 1913 (62.89) | 59.24 (11.66) | 52.72 (16.88) | yes | European | HDL associated PCs | 801 individuals |
| LBC1936 | 910 (50.2) | 69.68 (0.76) | 58.60 (16.92) | no | European | MDS 1-4 | 262 individuals |
| Lifelines | 12573 (41.66) | 49.14 (11.49) | 55.83 (14.93) | yes | European | pc1-10 | ~983 individuals |
| LLS | 2282 (45.53) | 59.19 (6.82) | 55.69 (17.51) | no | European | family relationships (QT-assoc) | 172 individuals |
| NTR-NESDA | 7564 (36.50) | 42.41 (14.51) | 56.7 (15.65) | yes | European | PC’s | none |
| PREVEND | 3574 (51.6) | 49.65 (12.49) | 50.62 (15.37) | yes | European | PC1-5 | 130 individuals |
| PROSPER | 5244 (48.10) | 75.34 (3.35) | 49.58 (13.53) | yes | European | PC1-4 | none |
| QIMR | 9014 (58) | 47.1 (12.5) | 58.5 (16.3) | no | European | none | none |
| TRAILS | 993 (52.42) | 16.20 (0.66) | 56.04 (11.50) | yes | European | pc1-10 | none |
| YFS | 2097 (45.02) | 31.72 (4.98) | 49.83 (12.24) | yes | European | study center, HDL associated PCs | 7 individuals |

**Supplemental table 2a.** SNP genotyping and imputation details of the discovery cohorts.

MAF = minor allele frequency; HWE = Hardy-Weinberg equilibrium

| **Cohort** | **chip** | **QC** | **Reference panel for imputations** | **Tool used for imputations** |
| --- | --- | --- | --- | --- |
| AGES | Illlumina Hu370CNV | MAF >0.01, >97% complete, HWE >1e-06 | 1000 Genomes project reference panel (version Phase 1 integrated release v3) | MaCH and Minimac |
| ARIC (AA) | Affy 6.0 | MAF >0.01, >95% complete, HWE >0.00001 | 1000 Genomes project reference panel (version Phase 1 integrated release v3). | Shapelt (v1.r532) and IMPUTE2 |
| ARIC (EA) | Affy 6.0 | MAF >0.005, >95% complete, HWE >0.00001 | 1000 Genomes project reference panel (version Phase 1 integrated release v3). | Shapelt (v1.r532) and IMPUTE2 |
| CHS (EA) | Illumina 370CNV and CARe IBC | Genotypes were called using the Illumina [BeadStudio](http://wildebeest.pbworks.com/BeadStudio) software. Samples were excluded from analysis for sex mismatch, discordance with prior genotyping, or call rate < 95%. The following exclusions were applied to identify a final set SNPs for imputation: call rate < 97%, HWE P < 10^-5^, > 2 duplicate errors or Mendelian inconsistencies (for reference trios), heterozygote frequency = 0. | 1000G phase 1 version 3 (all ancestries) | MaCH and Minimac |
| CROATIA-KORCULA | Illumina HumanHap370CNV | Individual call rate > 0.97, SNP call rate > 0.98, MAF >0.01, HWE > 1E-06 | 1000 Genomes project reference panel (version Phase 1 integrated release v3, march 2012, all populations). | SHAPEIT2 and IMPUTE2 |
| CROATIA-SPLIT | Illumina HumanHap370CNV | Individual call rate > 0.97, SNP call rate > 0.98, MAF >0.01, HWE > 1E-06 | 1000 Genomes project reference panel (version Phase 1 integrated release v3, march 2012, all populations). | SHAPEIT2 and IMPUTE2 |
| CROATIA-VIS | Illumina Infinium HumanHap300v1 | Individual call rate > 0.97, SNP call rate > 0.98, MAF >0.01, HWE > 1E-06 | 1000 Genomes project reference panel (version Phase 1 integrated release v3, march 2012, all populations). | SHAPEIT2 and IMPUTE2 |
| ERF | various Illumina and Affymetrix chips | callrate > 0.98, per individual callrate > 0.96, HWE *p*-value > 5 · 10^-8^ and MAF > 0.005. IBS, sex chromosome and ethnicity checks were also performed. | 1000 Genomes project reference panel (version Phase 1 integrated release v3, march 2012, all populations). | MaCH (1.0.18c) and Minimac (minimac-beta-2012-03-14). |
| FHS | Affymetrix 500K and MIPS 50K combined | BRLMM calling; Sample callrate > 0.97; SNP callrate > 0.97; HWE p value >10^-6^; for imputation MAF > 0.01 and Mendelian errors <1000 | 1000 Genomes project reference panel (version Phase 1 integrated release v3, april 2012, all populations | Minimac versions released 2012-05-29 and 2012-08-15 |
| FamHS | ILLUMINA 550K, ILLUMINA 610K, and ILLUMINA 1M chips | callrate > 0.98, per individual callrate > 0.98, HWE p-value > 1E-06 and MAF > 0.01. Mendelian errors, familial relationships based on IBS, sex and ethnicity checks were also performed | Cosmopolitan panel (including all races - version 2010-11 data freeze, 2012-03-04 haplotypes) | MaCH (1.0.18c) and Minimac (minimac-beta-2012-03-14). |
| Generation Scotland | Illumina OMNI Express + Exome | OMNI chip - Individual call rate > 0.97, SNP call rate > 0.98, MAF >0.01, HWE > 1E-06  Exome chip - Individual call rate > 0.99, SNP call rate > 0.98, MAF >0.0001, HWE > 1E-06 | 1000 Genomes project reference panel (version Phase 1 integrated release v3, march 2012, all populations). | SHAPEIT2 and IMPUTE2 |
| JHS | Affy 6.0 | SNP level callrate > 90%, sample level callrate >95%, MAF >0.01, HWE > 1E-06 | 1000 Genomes project reference panel (version Phase 1 integrated release v3, March 2012, all populations). | MACH and Minimac |
| MESA (AFA) | Affy 6.0 | SNP level callrate > 95%, sample level callrate > 95%, heterozygosity < 53%, MAF > 0 (remove monomorphic SNPs). | 1000 Genomes project reference panel (version Phase 1 integrated release v3, march 2012, all populations). | IMPUTE v2.2.2 |
| MESA (CAU) | Affy 6.0 | SNP level callrate > 95%, sample level callrate > 95%, heterozygosity < 53%, MAF > 0 (remove monomorphic SNPs). | 1000 Genomes project reference panel (version Phase 1 integrated release v3, march 2012, all populations). | IMPUTE v2.2.2 |
| MESA (CHN) | Affy 6.0 | SNP level callrate > 95%, sample level callrate > 95%, heterozygosity < 53%, MAF > 0 (remove monomorphic SNPs). | 1000 Genomes project reference panel (version Phase 1 integrated release v3, march 2012, all populations). | IMPUTE v2.2.2 |
| MESA (HIS) | Affy 6.0 | SNP level callrate > 95%, sample level callrate > 95%, heterozygosity < 53%, MAF > 0 (remove monomorphic SNPs). | 1000 Genomes project reference panel (version Phase 1 integrated release v3, march 2012, all populations). | IMPUTE v2.2.2 |
| ORCADES | Illumina Hap300, Omni1, OmniX | Callrate >97%, per individual callrate>98%, HWE p-value >10^-6^ , monomorphic SNPs, MAF >0.001, ethnic outliers, duplicates, gender mismatch, excess IBS or Mendelian inconsistency incompatible with pedigree | 1000 Genomes Phase 1 integrated release version 3 haplotypes | IMPUTE v2.2.2 |
| RS-I | Illumina 550K | MAF < 0.05, SNP callrate < 0.95 and/or HWE *p*-value < 1 · 10^-7^ | 1000 Genomes project reference panel (version Phase 1 integrated release v3, march 2012, all populations). | MaCH and Minimac |
| RS-II | Illumina 550K | MAF < 0.05, SNP callrate < 0.95 and/or HWE *p*-value < 1 · 10^-7^ | 1000 Genomes project reference panel (version Phase 1 integrated release v3, march 2012, all populations). | MaCH and Minimac |
| RS-III | Illumina 610K and 660K | MAF < 0.05, SNP callrate < 0.95 and/or HWE *p*-value < 1 · 10^-7^ | 1000 Genomes project reference panel (version Phase 1 integrated release v3, march 2012, all populations). | MaCH and Minimac |

**Supplemental table 2b.** SNP genotyping and imputation details of the replication cohorts.

| **Cohort** | **chip** | **QC** | **Reference panel for imputations** | **Tool used for imputations** |
| --- | --- | --- | --- | --- |
| Athero-Express | Affymetrix SNP 5.0 (Athero-Express Genomics Study 1); Affymetrix Axiom CEU (Athero-Express Genomics Study 2) | Genotypes were called using Affymetrix’ GCOS with the default settings. Samples were excluded from analysis for sex mismatch, IBS, and PCA, or call rate < 85% (AEGS1) or < 95% (AEGS2). SThe following exclusions were applied to identify a final set SNPs for imputation: call rate < 97%, HWE P < 10^-6^, MAF<3%. | 1000G phase 1 version 3 (all ancestries) | IMPUTE V2 |
| CHS (AA) | Illumina Omni1M | Genotypes were called using the Illumina [BeadStudio](http://wildebeest.pbworks.com/BeadStudio) software. Samples were excluded from analysis for sex mismatch, discordance with prior genotyping, or call rate < 95%. The following exclusions were applied to identify a final set SNPs for imputation: call rate < 97%, HWE P < 10^-5^, > 2 duplicate errors or Mendelian inconsistencies (for reference trios), heterozygote frequency = 0. | 1000G phase 1 version 3 (all ancestries) | IMPUTE V2 |
| FINCAVAS | Metabochip | callrate > 0.98, per individual callrate > 0.95, HWE *p*-value > 5 · 10^-6^ and MAF > 0.01. IBS, sex chromosome and heterozygosity checks were also performed. | 1000 Genomes project reference panel (version Phase 1 integrated release v3, March 2012, all populations). | SHAPEIT (v2.r644) and IMPUTE (v2.3.0) |
| LBC1936 | Illumina 610-Quadv1 | SNP callrate > 0.98, sample callrate > 0.95, HWE *p*-value > 10^-3^ and MAF > 0.01. Relatedness and ethnicity checks were also performed | 1000 Genomes project reference panel (Phase 1 v3 March 2012 ALL) | Minimac |
| Lifelines | Illumina Cyto SNP12 v2 | SNP QC: callrate > 0.95, HWE *p*-value > 10^-4^, MAF > 0.001. Sample QC: callrate > 0.95, heterozygosity (<4SD from mean), IBS<0.35, sex match with phenotype, caucasian. | 1000 Genomes project reference panel (version Phase 1 integrated release v3, March 2012, all populations). | Minimac v2012.10.3 |
| LLS | Illumina Human660W / Illumina OmniExpress | callrate > 0.95, per individual callrate > 0.95, HWE *p*-value > 1 · 10^-4^ and MAF > 0.01. IBS and sex chromosome checks were also performed. | 1000 Genomes project reference panel (version Phase 1 integrated release v3, March 2012, all populations). | IMPUTE (v2.2) |
| NTR-NESDA | various Illumina and Affymetrix chips | MAF < 0.01, HWE *p*-value < 1 · 10^-5^ and call rate < 0.95, Samples were excluded in case of sex mismatch, genotype missing rate > 0.1 or Plink F inbreeding value was either > 0.10 or < -0.10 (heterozygosity). Imputation quality cutoff R^2^ < 0.30 | 1000 Genomes project reference panel (version Phase 1 integrated release v3, March 2012, all populations). | MaCH (version 1.0.18) and Minimac (version 2012.10.9 beta) |
| PREVEND | Illumina CytoSNP12 v2 | Individuals were excluded based on callrate <95%, etnicity (PC outliers), IBS, sex inconsitencies, exclusion of SNPs with Callrate <95% and pHWE<10E-6) | 1000 Genomes project reference panel (version Phase 1 integrated release v3, April 2012, all populations). | Shapeit and Impute2 |
| PROSPER | Illumina 660K Beadchip | Callrate >97,5%, HWE p-value 1.0x10^-6^. IBS, sex and ethnicity checks were also performed. | 1000 Genomes project reference panel (version Phase 1 integrated release v3, March 2012, all populations). | Impute |
| QIMR | Illumina (317K, 370K, 610K) | SNPs were excluded if call rate < 95%, HWE p < 10^-6^, MAF < 0.01. Subjects were excluded for Mendelian errors or non-European ancestry. | 1000G release of August 4^th^ 2010 | Mach and minimac |
| TRAILS | Illumina Cyto SNP12 v2 | SNP QC: callrate > 0.95, HWE *p*-value > 10^-4^, MAF > 0.01. Sample QC: callrate > 0.95, heterozygosity (<4SD from mean), IBS<0.35, sex match with phenotype, caucasian. | 1000 Genomes project reference panel (version Phase 1 integrated release v3, March 2012, all populations). | IMPUTE v2.2.2 |
| YFS | Illumuna 670k custom | callrate > 0.95, per individual callrate > 0.95, HWE *p*-value > 5 · 10^-6^ and MAF > 0.01. IBS, sex chromosome, cryptic relatedness and heterozygosity checks were also performed. | 1000 Genomes project reference panel (version Phase 1 integrated release v3, march 2012, all populations). | SHAPEIT (v1) and IMPUTE (v2.2.2) |

**Supplemental table 3.** Linkage disequilibrium (r^2^) between the five independent variants and rs3764261 within the *CETP* region using the 1000 Genomes data (1000G Phase I Integrated Release Version 22 Haplotypes (2010-11 data freeze, 2012-02-14 haplotypes)).

|  | rs3764261 | rs12920974 | rs34065661 | rs5817082 | rs4587963 | rs7499892 |
| --- | --- | --- | --- | --- | --- | --- |
| rs3764261 | - | 0.147 | 0.018 | 0.007 | 0.104 | 0.001 |
| rs12920974 |  | - | 0.052 | 0.010 | 0.095 | 0.042 |
| rs34065661 |  |  | - | 0.067 | 0.005 | 0.002 |
| rs5817082 |  |  |  | - | 0.161 | 0.467 |
| rs4587963 |  |  |  |  | - | 0.058 |
| rs7499892 |  |  |  |  |  | - |

**Supplemental table 4. The start and end positions of the exons of the *CETP* gene.**

| **Exon** | **Exon Chr Start (bp)** | **Exon Chr End (bp)** |
| --- | --- | --- |
| 1 | 56,995,762 | 56,996,009 |
| 2 | 56,996,922 | 56,997,036 |
| 3 | 57,003,298 | 57,003,432 |
| 4 | 57,003,523 | 57,003,593 |
| 5 | 57,003,826 | 57,003,913 |
| 6 | 57,004,945 | 57,005,014 |
| 7 | 57,005,233 | 57,005,293 |
| 8 | 57,005,904 | 57,005,995 |
| 9 | 57,007,243 | 57,007,422 |
| 10 | 57,009,013 | 57,009,063 |
| 11 | 57,012,003 | 57,012,167 |
| 12 | 57,015,070 | 57,015,137 |
| 13 | 57,015,559 | 57,015,592 |
| 14 | 57,016,077 | 57,016,149 |
| 15 | 57,017,238 | 57,017,323 |
| 16 | 57,017,504 | 57,017,757 |
